# Low-level resource partitioning supports coexistence among functionally redundant bacteria during successional dynamics

**DOI:** 10.1093/ismejo/wrad013

**Published:** 2024-01-10

**Authors:** Xiaoqian Annie Yu, Craig McLean, Jan-Hendrik Hehemann, David Angeles-Albores, Fuqing Wu, Artur Muszyński, Christopher H Corzett, Parastoo Azadi, Elizabeth B Kujawinski, Eric J Alm, Martin F Polz

**Affiliations:** Department of Biology, Massachusetts Institute of Technology, Cambridge, MA 02139, United States; Division of Microbial Ecology, Department of Microbiology and Ecosystems Science, Centre for Microbiology and Environmental Systems Science, University of Vienna, Vienna 1030, Austria; Department of Marine Chemistry and Geochemistry, Woods Hole Oceanographic Institution, Woods Hole, MA 02543, United States; MIT/WHOI Joint Program in Oceanography/Applied Ocean Science and Engineering, Department of Marine Chemistry and Geochemistry, Woods Hole Oceanographic Institution, Woods Hole, MA 02543, United States; Department of Civil and Environmental Engineering, Massachusetts Institute of Technology, Cambridge, MA 02139, United States; Department of Biological Engineering, Massachusetts Institute of Technology, Cambridge, MA 02139, United States; Department of Biological Engineering, Massachusetts Institute of Technology, Cambridge, MA 02139, United States; Complex Carbohydrate Research Center, University of Georgia, Athens, GA 30602, United States; Department of Civil and Environmental Engineering, Massachusetts Institute of Technology, Cambridge, MA 02139, United States; Complex Carbohydrate Research Center, University of Georgia, Athens, GA 30602, United States; Department of Marine Chemistry and Geochemistry, Woods Hole Oceanographic Institution, Woods Hole, MA 02543, United States; Department of Civil and Environmental Engineering, Massachusetts Institute of Technology, Cambridge, MA 02139, United States; Department of Biological Engineering, Massachusetts Institute of Technology, Cambridge, MA 02139, United States; Broad Institute of MIT and Harvard, Cambridge, MA 02139, United States; Center for Microbiome Informatics and Therapeutics, Massachusetts Institute of Technology, Cambridge, MA 02139, United States; Division of Microbial Ecology, Department of Microbiology and Ecosystems Science, Centre for Microbiology and Environmental Systems Science, University of Vienna, Vienna 1030, Austria; Department of Civil and Environmental Engineering, Massachusetts Institute of Technology, Cambridge, MA 02139, United States

**Keywords:** functional redundancy, resource partitioning, coexistence, community assembly

## Abstract

Members of microbial communities can substantially overlap in substrate use. However, what enables functionally redundant microorganisms to coassemble or even stably coexist remains poorly understood. Here, we show that during unstable successional dynamics on complex, natural organic matter, functionally redundant bacteria can coexist by partitioning low-concentration substrates even though they compete for one simple, dominant substrate. We allowed ocean microbial communities to self-assemble on leachates of the brown seaweed *Fucus vesiculosus* and then analyzed the competition among 10 taxonomically diverse isolates representing two distinct stages of the succession. All, but two isolates, exhibited an average of 90% ± 6% pairwise overlap in resource use, and functional redundancy of isolates from the same assembly stage was higher than that from between assembly stages, leading us to construct a simpler four-isolate community with two isolates from each of the early and late stages. We found that, although the short-term dynamics of the four-isolate communities in *F. vesiculosus* leachate was dependent on initial isolate ratios, in the long term, the four isolates stably coexist in *F. vesiculosus* leachate, albeit with some strains at low abundance. We therefore explored the potential for nonredundant substrate use by genomic content analysis and RNA expression patterns. This analysis revealed that the four isolates mainly differed in peripheral metabolic pathways, such as the ability to degrade pyrimidine, leucine, and tyrosine, as well as aromatic substrates. These results highlight the importance of fine-scale differences in metabolic strategies for supporting the frequently observed coexistence of large numbers of rare organisms in natural microbiomes.

## Introduction

Although microbial communities typically harbor extensive taxonomic diversity, the number of functions encoded within the sum of coexisting genomes appears to be far less [1, 2]. This observation has highlighted that many organisms encode similar functions and are thus considered to be functionally redundant, and the degree of functional niche overlap among taxa is often used as an important measurement for evaluating the amount of functional redundancy within a community [1, 3]. Because this implies that the community members are at least to some extent replaceable, functional redundancy is thought to allow for more resilience toward biotic and abiotic perturbations [4-6]. That is, even if taxa fluctuate, important “core” functions at the ecosystem level remain invariant. These core functions reflect the habitat in which a community assembles [7, 8], and while they may appear to be stable within a habitat, taxa can show high turnover [9] in support of the notion that redundancy can preserve the overall ecosystem services provided by microbial communities.

High functional redundancy is seemingly at odds with classical competition theory, which allows only for one taxon to persist for each limiting resource in a well-mixed, steady-state community [10, 11]. Therefore, to stably coexist, functionally redundant organisms must be differentiated in additional niches. Indeed, temporal and spatial resource partitioning are primary mechanisms that foster coexistence within habitats. For example, following phytoplankton blooms in the ocean, sympatric bacteria transiently bloom over time, alleviating their competition for algal polysaccharides such as starch and laminarin [12, 13]. Similarly, bacteria also minimize resource competition by segregating spatially on different nutrient patches in the gut [14], plant roots [15], or marine particles [16, 17], either due to metabolic specialization or different dispersal and mobility strategies [18-20]. In well-mixed systems lacking spatial and temporal structures, metabolite cross-feeding [21] and fitness differences on shared substrates [22], chemical warfare [23] as well as trade-offs between fast growth and shorter diauxic lags [24] have all been shown to support the coexistence of functionally redundant bacteria in simple one- or two-substrate systems. Finally, one recent study demonstrated that four *Lactobacillus* species isolated from the bee could coexist on the glucose in pollen because they were able to differentially utilize the minor (low concentration) nutrients present in pollen [25]. Because the latter results suggest that low-level resource partitioning, i.e. differential use of low-concentration substrates, may be sufficient for coexistence, we were interested whether a similar situation might arise in the inherently unstable coastal ocean system that is characterized by rapid successions due to algal blooms [26, 27] and where coexistence has been previously primarily ascribed to temporal and spatial resource partitioning [12, 13, 17, 28].

Here, we provide an experimental demonstration of how highly functionally redundant bacteria can stably coexist during different stages of an inherently dynamic succession. We constructed a model system with isolates originating from the self-assembly process of natural marine communities on the leachate of the brown seaweed *Fucus vesiculosus* (FL) in microcosms. This model system illustrates the importance of fine-scale resource partitioning, i.e. the partitioning of multiple nutrients that exist at small amounts, for the consistent coassembly of bacteria in successions.

## Results

### Succession of natural communities on *F. vesiculosus* leachate

To study the successional dynamics of marine microbial communities under laboratory but realistic conditions, we developed a model system that consists of batch cultures of minimal seawater media where the carbon source consisted of leachate from dead fronds of the brown seaweed *F. vesiculosus*. This situation is analogous to the first stages in the decay process of dead seaweed and represents an important process in many coastal environments [29]. Glycosyl composition analysis of FL demonstrated that mannitol was the major carbohydrate constituent of the leachate (86.9% of carbohydrate, 28.7% total dry weight; Fig. S1a–c). The remaining 13.1% of the detected carbohydrates was a mixture of polysaccharides, which, based on the monomer composition and their glycosyl linkage, likely included fucoidan, alginate, and laminarin (Fig. S1c and d). The H^1^-NMR analysis also suggested that, in addition to glycosyl components, the substrate consists of aromatic compounds as well as amine-containing compounds such as protein (Fig. S1e). We reasoned that such a system with a complex and environmentally relevant carbon substrate, but no apparent spatial structure, allows for studying the metabolic rules involved in community assembly, such as how the metabolic self-organization of complex communities could be driven by cross-feeding, differential use of minor substrates that are present in small amounts, or relative strength of competition on various shared substrates. Furthermore, the system is well suited to study how the differences in resource utilization could affect community assembly because the dominant component of FL (mannitol) is widely utilized by marine bacteria, while metabolisms of less-abundant compounds in FL are more restricted.

**Figure 1:**
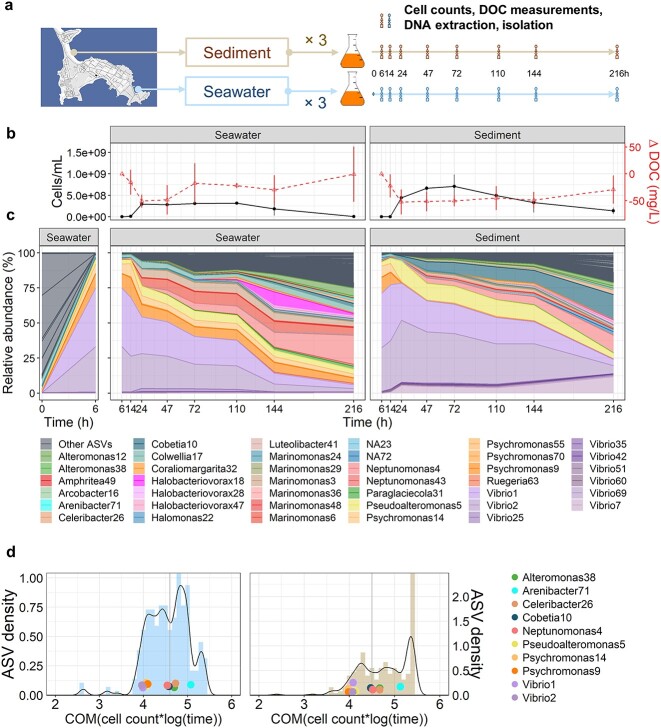
Microbial community successions on brown macroalgal leachate; (A) experimental setup for studying the succession of natural microbial communities on leachate of the brown seaweed *F. vesiculosus* (FL); left: map of Nahant [58] showing the sampling locations of the seawater and sediment communities used as inocula (scale: 1:10 000); right: schematic of the self-assembly process on FL; inoculant communities were grown in FL batch media for 216 h, and at each time point between 6 and 216 h, cell count and DOC measurements were taken; community composition was determined in the inocula and at each time point by 16S rRNA gene tag sequencing; ~30–40 isolates were collected from each replicate community at each time point; (B) average trajectory of total bacterial abundances (as cell density) and the change in concentration of DOC relative to *t* = 6 h (negative values = decrease of DOC) in the seawater- and sediment-inoculated communities over time; error bars are standard deviations (*n* = 3); (C) community composition over time shown as the relative abundances of taxa averaged over three replicates from seawater- and sediment-inoculated communities; the top 40 most abundant taxa (amplified sequence variants, ASVs) are shown in color; the sum of other ASVs is shown in gray; the community composition changes from 0 to 6 h in the seawater-inoculated communities are in a separate panel on the left to emphasize the change from the inoculum to the first bloom; (D) distribution of the COM, $\sum [\mathrm{Cell}\ \mathrm{Density}\times \log (\mathrm{time})]/\sum \mathrm{Cell}\ \mathrm{Density}$) for the absolute abundance of individual taxa in the seawater- (blue, left) and sediment- (brown, right) inoculated communities; distributions are represented as both histograms (colored bars) and kernel density estimations (black lines); absolute abundances are calculated as the product of community cell density in (B) with relative abundances in (C); gray lines at the first trough of density plots represents the cutoff between early and late growers in the successions; colored dots represent the five selected representative ASVs from early and late bloomers, respectively.

To initiate the community assembly process, we inoculated the system in triplicate with two environmental samples collected from the free-living (0.2–5 μm) fraction of coastal surface ocean seawater and from particles obtained from a shallow sediment. We then sampled the system at discrete time intervals to quantify the changes in cell density and amount of dissolved organic carbon (DOC) and assessed community composition changes using 16S rRNA gene amplicon sequencing. Isolates were also collected at each time point for genomic and metabolic characterizations (Fig. 1a).

Despite their distinct origin, the two natural communities exhibited similar changing patterns in the bulk community function and overall community turnover on the FL: both the seawater- and sediment-inoculated systems experienced initial rapid increases in the total cell density, subsequent stabilization, and eventual slow decline (Fig. 1b). In accordance with the changes in cell density, the amount of DOC in the system first rapidly decreased, but then gradually recovered (Fig. 1b). This suggests that the early responding taxa undergo boom and bust dynamics: their initial fast expansion in population is followed by cell death and release of cellular contents into the system. Consistent with this interpretation, we observed a strong change in community composition between 0 and 6 h (Fig. 1c) as well as a substantial increase of the amplicon sequencing variants (ASVs) representative of the predatory bacteria *Halobacteriovorax* in the later time points (Fig. 1c). While both communities were nearly completely dominated by *Vibrio* and *Psychromonas* ASVs at early stages (6–14 h), the seawater- and sediment-inoculated systems diverged in later time points (Fig. S2) because of a second bloom (starting at 24 h) consisting of partially different ASVs in the two systems (Fig. 1c). Thus, the assembly processes of the seawater and sediment communities in the FL model system show hallmarks of ecological succession, where an orderly replacement of species happens over time accompanied by changes of the physical environment.

Because the DOC and cell number dynamics as well as community turnover suggested at least two growth phases in the community succession, we established a quantitative criterion for determining whether an ASV is an “early” or a “late” bloomer using the center of mass (COM) of its absolute growth trajectory. Consistent with the observed successional dynamics, in both the seawater- and sediment-inoculated communities, the distribution of ASV COMs was multimodal, indicating that there were at least three groups of bacteria in each community that peaked at different times (Fig. 1d). Furthermore, ASVs within the same taxonomic family were more likely to follow the same absolute growth trajectories compared to ASVs from different families (Wilcoxon rank-sum test, *P* < 2.2 × 10^−16^, Fig. S3), indicating that many of the mechanisms underlying the community assemblies are conserved at the family level.

### Model community construction

We investigated whether basic features of the successional dynamics could be reconstituted with a model consortium that represents the taxonomic diversity as well as abundant ASVs from both the early and late bloom groups. Using the isolates collected from the successions, we were able to match five ASVs from each of the two groups with isolates that were identical in the 16S rRNA V4 region and were drawn from eight taxonomic families (Fig. 1d). Together, the five isolates that represent the early ASVs cover 98.9% and 92.4% of the sequencing reads at early timepoints (*t* = 14 h) in the seawater- and sediment-inoculated communities, respectively. At the last time point (*t* = 216 h), the five isolates representing the late ASVs cover 35.3% and 35.0% of reads in the seawater- and sediment-inoculated communities, respectively. Further, considering that the ASV growth trajectories are similar at the family level (Fig. S3), the five late isolates represent families that cover 71.1% (seawater) and 51.6% (sediment) of the reads at the last time point. After mixing the 10 selected isolates in abundances similar to the natural seawater inoculum (Supplementary Methods) and observing the community growth and composition change over 216 h, we found that this simple isolate-constituted model community was less affected by predation compared to the natural community (Fig. S4a), and the metabolic differences between the selected bacteria are sufficient to consistently sustain a similar succession pattern to that of the natural community-inoculated microcosms (Fig. S4b and c).

### Isolates within the model community are highly functionally redundant

**Figure 2:**
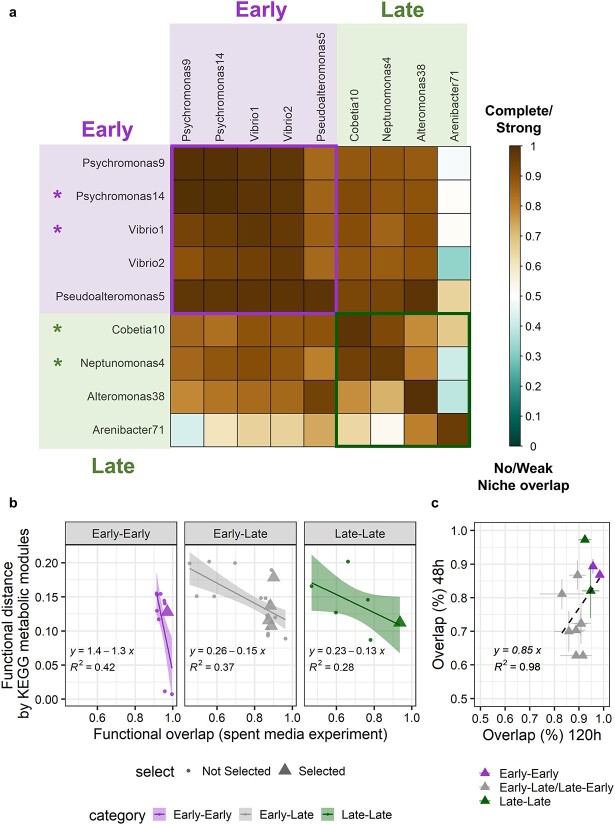
Spent-media experiments reveal high levels of functional redundancy among representative isolates; (A) proportional growth of each isolate (rows) in the spent-media (120 h) of another isolate (columns); the color of each square represents overlap of resource use between two isolates, in the proportion of reduced growth compared to growth in fresh FL media, on a scale of 0–1; early isolates (top left box) are represented with purple background color, and late isolates (bottom right box) are represented with green background color; isolates marked with ^*^ were further selected for the four-isolate model community; (B) relationship between pairwise FD of nine isolates estimated via metabolic pathway (KEGG module) distributions in genomes and average pairwise functional overlap measured via spent-media experiments; isolate pairs were divided into three categories depending on whether they belonged to the same early or late assembly group; isolate pairs that represent the final four-isolate model community are represented in triangles; (C) functional overlap measured in 48 h versus 120 h spent-media experiments for the strains in the four-isolate model community; for each pair of isolates, reciprocal functional overlaps (Isolate 1 growing in spent-media of Isolate 2, and vice versa) are shown; error bars represent standard deviations (*n* = 3).

Because we hypothesized that the observed successional dynamics are due to metabolic differentiation, we set up a series of experiments to determine to what extent isolates within and between the early and late groups are functionally differentiated. We first performed a Burkholder diffusion assay (Supplementary Methods) to test for the potential of interference competition between our isolates [30, 31], and we found that, with the exception of Celeribacter26, interference between our isolates was rare and weak (63/72, no interference; 9/72, weak interference; Fig. S5). Furthermore, because the Burkholder assay is performed using bacterial lawns at ~40× substrate concentration of the microcosms, it is likely that the few weak interactions observed in the Burkholder assay are negligible in our microcosms. Based on these results, we excluded Celeribacter26 from further experiments and used spent-media experiments to quantitatively assess the metabolic overlap of the nine remaining isolates as well as to select a further subset for detailed metabolic characterization. We compared the maximum cell density of each isolate when grown to late stationary phase (120 h) in spent-media of all other isolates versus fresh FL media. This ratio allows direct evaluation of the extent of metabolic functional redundancy (i.e. overlap in resource use) between the nine isolates (see Methods, Fig. 2a and Fig. S6).

We found that the functional redundancy between isolates was generally high, even for distantly related bacteria. When not accounting for the single outlier Arenibacter71, the average overlap in resource use between any pair of isolates was 90% ± 6.3%. Isolates that belong to the early group showed an even stronger (94% ± 5.1%) overlap in resource use, whereas the late group was slightly more heterogeneous with a strong overlap between Cobetia10 and Neptunomonas4 (94% ± 1.7%), and these two isolates having much less overlap with Alteromonas38 (77% ± 3.6%) and Arenibacter71 (56% ± 12%). All isolates in the late group also showed some growth on the spent-media of the early group isolates, explaining their later growth in the succession (Fig. 2a). They were either capable of using resources that were not taken up by the early isolates or can feed on their metabolic byproducts. Thus, although there was widespread functional redundancy in our model community, small differences in the substrate utilization between individual isolates may have been enough to allow for the coexistence during succession dynamics in the community.

We next evaluated how the phenotypic differences in resource utilization between the isolates relate to the metabolic potential inferred from their genomes (Methods). This comparison showed that functionally more distant isolates based on the prediction of KEGG modules also showed less overlap in the spent-media experiments (Fig. 2b). The early isolates were higher in resource use overlap and had an overall more negative slope (slope = −1.3) in their genotype–phenotype relationship than the late isolates (slope = −0.13). This difference is primarily driven by the greater spread in phenotypic overlap in the spent-media and suggests that the late isolates were likely capable of using a broader set of substrates compared to the early isolates.

Based on the functional redundancy information, we further simplified the model community to four isolates. Using the criteria of large, but not complete, functional overlap within each of the two assembly groups, and resource use overlap within each assembly group being higher than between assembly groups, Psychromonas14 and Vibrio1 were selected as representatives of the early group and Cobetia10 and Neptunomonas4 were selected as representatives of the late group (Supplementary Methods, Fig. 2b and c). The resource overlap among these isolates was consistent when grown for shorter periods (48 vs. 120 h), suggesting that more labile substrates, which are used early on, are primarily responsible for the high potential redundancy (Fig. 2c).

**Figure 3:**
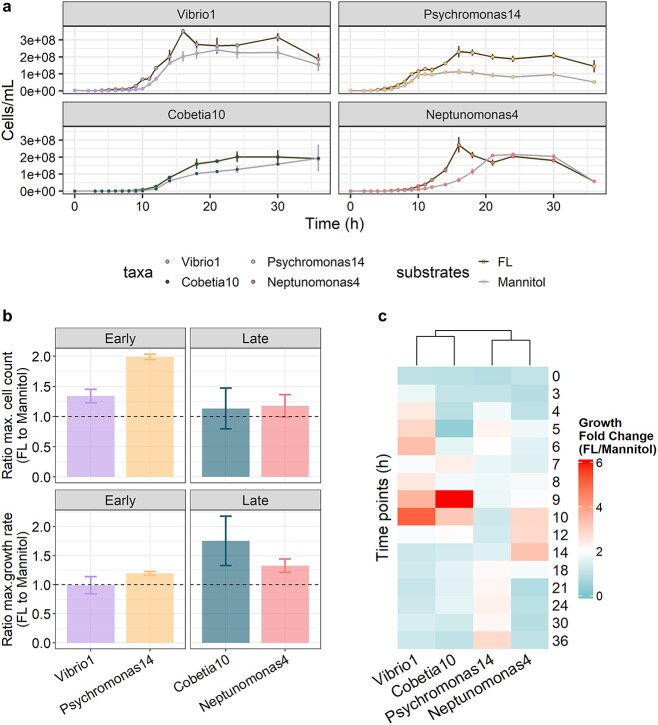
Growth patterns of isolates in FL and mannitol confirm high functional redundancy; (A) growth curves of the four model strains in FL (dark gray) and mannitol (0.0047% w/v, matching the mannitol concentration in FL, light gray) media as the only carbon source; (B) top: ratio of maximum carrying capacity, measured as the maximum cell count reached at any time point within 0–36 h of growth, for the four strains grown in FL versus mannitol media, separated by early and late groups; bottom: ratio of maximum per capita growth rate, measured as the maximum segment linear growth rate for at least four consecutive time points within 0–12 h of growth, for the four strains grown in FL versus mannitol media, separated by early and late groups; (C) heatmap for ratio of cell density in FL versus mannitol media for each time point during 0–36 h of growth for the four strains; strains with similar growth ratio changes over time were clustered together with hierarchical clustering using the complete linkage method.

Because all strains can utilize mannitol, which constitutes the major labile carbon source in the algal leachate, we sought to quantify to what extent other carbon sources might contribute to the fitness of each strain in the FL media. We therefore compared the growth curves of the isolates in the FL media to that in media with mannitol as the only carbon source by providing mannitol at equal concentration to that estimated in FL media (0.0047% w/v) (Fig. 3a). Although the growth in the FL media appears to be more complex, the data suggest that mannitol was indeed the major source of growth, supporting between 70% and 100% of the cell growth for Vibrio1, Cobetia10, and Neptunomonas4 as well as ~50% of the cell growth for Psychromonas14 (Fig. 3b, top panel). Moreover, growth curves for Vibrio1 and Psychromonas14 in FL were characteristic of diauxic growth with a short lag phase between the two fast growth phases, and the maximum growth rate of Vibrio1 during the first growth phase in FL was not significantly different from that in mannitol (ratio *t*-test, *P* = .87, Fig. 3b, bottom panel), while for Psychromonas14, the growth rate during the first phase in FL was slightly higher than that in mannitol (ratio *t*-test, *P* = .041, Fig. 3b, bottom panel). This suggests that the two isolates prefer mannitol compared to the other substrates available in FL, consistent with their assignment to the early assembly group. By contrast, we did not observe two-stage growth for the Cobetia10 and Neptunomonas4 isolates, and their maximum growth rate in FL was significantly higher than that in mannitol (ratio *t*-test, *P* = .0074 and *P* = .033, Fig. 3b, bottom panel), indicating that these isolates may have been utilizing other substrates in the media simultaneously with mannitol. A further detailed comparison of the FL and mannitol growth curves at each time point reveals that organisms in the same assembly group show distinct periods of faster growth in FL compared to mannitol (Fig. 3c), suggesting they may have different preferences and utilization sequences for the non-mannitol substrates in FL. Furthermore, Arenibacter71, one of the isolates we eliminated due to its low functional redundancy with the other isolates, was incapable of growing in mannitol (Fig. S7a). This inability is consistent with mannitol underpinning functional redundancy in the system, but this also indicates that there are other carbon sources in the FL that play a role.

### Coexistence in the four-isolate model system

We used the four-strain model system to further investigate if the coassembly of isolates within each assembly group was truly a result of resource partitioning, or if it is simply a result of them being introduced to the microcosm at similar initial concentrations. We found that, in the short term, the initial inoculation ratios of isolates could indeed alter the succession dynamics as well as lead to orders of magnitude differences in the abundance of a microbe (Fig. S8a and b). We therefore checked if, in the long term, the differently inoculated systems converge to the same steady state, because this would suggest that the coassembly of isolates during succession is at least partially due to a differential resource use and not solely a result of the initial inoculation ratios. We systematically varied the amounts of Vibrio1, Psychromonas14, Cobetia10, and Neptunomonas4 in the inoculum (Methods), and we propagated the communities through nine growth-dilution cycles in FL media and 0.0047% mannitol, respectively. In each cycle, cells were cultured for 48 h before being diluted into fresh media by a factor of 100, which corresponds to a total of ~60 generations, allowing the community to equilibrate to a stable composition.

**Figure 4:**
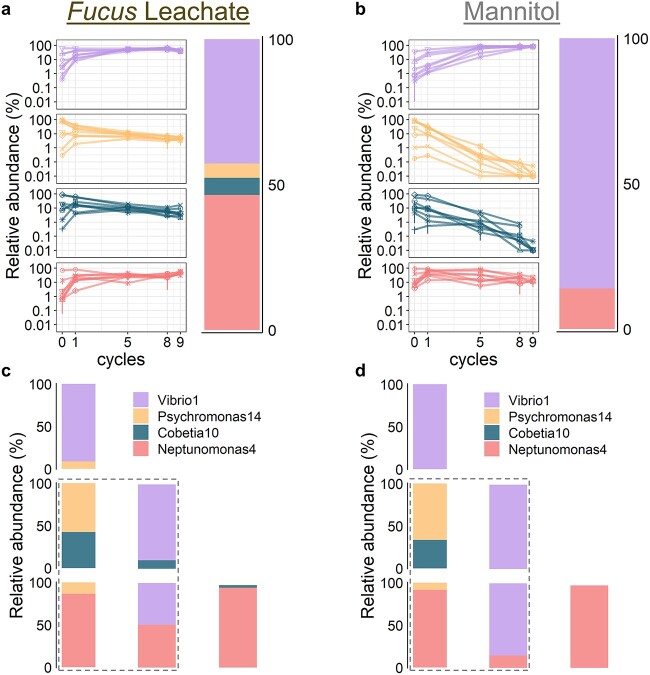
Stable coexistence in the four-isolate model system; (A) relative abundance trajectories of Psychromonas14, Vibrio1, Cobetia10, and Neptunomonas4 in eight representative sets of four-isolate competition experiments in *Fucus* leachate (FL, left, *n* = 3; error bars are standard deviation), and the average community composition at stable coexistence after nine cycles of 48 h growth (right, *n* = 8); (B) relative abundance trajectories of Psychromonas14, Vibrio1, Cobetia10, and Neptunomonas4 in eight representative sets of four-isolate competition experiments in mannitol (left, *n* = 3, error bars are standard deviation), and the average community composition at stable coexistence after nine cycles of 48 h growth (right, *n* = 8); (C) outcomes of pairwise competition in FL medium for Psychromonas14, Vibrio1, Cobetia10, and Neptunomonas4 (see Fig. S10 for changes in community composition over time); graphs in the gray dashed box represent communities containing isolates from different assembly groups; all graphs represent the average community composition of three replicate communities; **(**D) outcomes of pairwise competition in 0.0047% mannitol medium for Psychromonas14, Vibrio1, Cobetia10, and Neptunomonas4 (see Fig. S10 for changes in community composition over time); graphs in the gray dashed box represent communities containing isolates from different assembly groups; all graphs represent the average community composition of three replicate communities.

We found that, despite its apparently low level, resource partitioning among the bacteria could support their stable coexistence. Systems starting with a majority of Vibrio1, Psychromonas14, Cobetia10, or Neptunomonas4 resulted in final communities with stable coexistence of all four strains in FL (Fig. 4a and Fig. S9a). However, in mannitol, only Vibrio1 and Neptunomonas4 were able to stably coexist (Fig. 4b and Fig. S9b). The stable state of the four-isolate communities consists of 41.7% ± 5.1% Vibrio1 and 47.9% ± 7.2% Neptunomonas4, but only 4.4% ± 1.7% Psychromonas14 and 6.0% ± 4.2% Cobetia10. Since both Psychromonas14 and Cobetia10 go extinct when grown in mannitol only, this strongly suggests that, although on mannitol, the two organisms are less competitive compared to Vibrio1 and Neptunomonas4, on some other substrates in FL, they have a higher fitness and are more competitive. However, since Psychromonas14 and Cobetia10 each constitutes only ~5% of the equilibrium community, these other substrates must be much lower in abundance compared to mannitol; especially, if assuming a similar carbon-to-biomass conversion rate for all substrates, we can estimate these substrates to consist of ~10% of the total carbon utilized, while the other 90% is all mannitol.

We were able to further tease apart coexistence mechanisms in the four-strain community by utilizing pairwise competition experiments. All pairwise combinations of the four isolates were inoculated at initial ratios of 10:90 or 90:10 and were propagated through nine growth-dilution cycles in the same manner as in the previous four-isolate competitions. We found that, in FL media, all pairs converged to roughly the same ratio regardless of their initial inoculation ratios (*P* = 1 for all pairs, Fisher’s exact test; Fig. 4c for outcomes; Fig. S10a for trajectories). However, in mannitol, only isolates from the different assembly groups could stably coexist (*P* = 1 for all pairs, Fisher’s exact test): Vibrio1 outcompeted Psychromonas14 for the early group isolates, and Neptunomonas4 outcompeted Cobetia10 for the late group isolates (Fig. 4d for outcomes, Fig. S10b for trajectories). Importantly, the relative ratios of the strains in the four-member community was replicated in the pairwise combinations, indicating that the assembly process was mainly driven by pairwise interactions [32], and these pairwise interaction patterns were not modified by the presence of extra community members. Also, because our previous Burkholder diffusion assay showed that there was negligible antagonism between these four isolates, antagonism likely did not play a role in supporting the coexistence of the four isolates in FL.

Comparing the pairwise competition results in FL and mannitol, we infer that the coexistence between the early and late groups in FL was partially driven by cross-feeding. For example, Neptunomonas4 was able to grow in the Vibrio1 spent-mannitol-media (Fig. S11), and coexist in mannitol with Vibrio1, but the relative abundance of Neptunomonas4 to Vibrio1 at stable state was lower in mannitol compared to in FL (Fig. 4c and d). Thus, the additional growth of Neptunomonas4 in FL spent-media was likely supported by non-mannitol-derived substrates in FL. On the other hand, although isolates were sometimes able to grow on the spent-mannitol-media of isolates from the same assembly group (Fig. S11), the amount of cross-feeding was not sufficient to support the coexistence between the isolates in mannitol (Fig. 4d). Therefore, differences in using minor components in the FL media was the key driving force for the coexistence of isolates within each assembly group.

### Fine-scale metabolic differences in the four-isolate model system

Based on the above suggestion that coexistence is driven by the ability to use minor, low-concentration components in the FL media, we investigated what types of substrates might differentiate the four isolates in our FL model system. We took a three-step approach: first, in each assembly group, we identified the KEGG modules that were only complete in the genome for one of the two isolates, and we hypothesized that these-module related substrates were those being partitioned in the FL; we then narrowed down the range of the substrates by checking if the modules specific to each isolate are expressed during growth of the isolate in FL media; finally, we confirmed that our isolates had differential usage abilities for the module-associated substrates with growth assays of each isolate.

**Figure 5:**
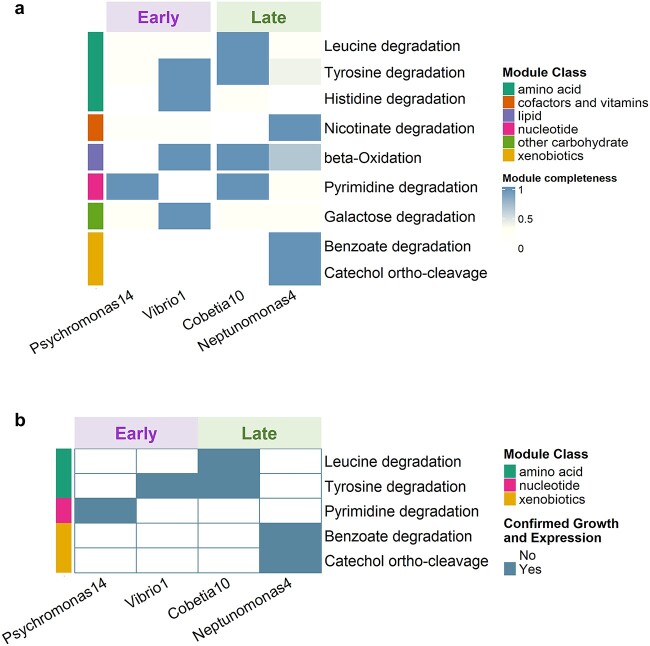
Potential mechanisms of niche partitioning in FL; (A) heatmap for all KEGG modules that had different genomic completeness for isolates within at least one assembly group; (B) heatmap for all KEGG modules that were expressed and growth confirmed and had different completeness for isolates within at least one assembly group.

We identified a total of nine KEGG modules that are associated with substrate degradation pathways and differed in genomic completeness within an assembly group (Fig. 5a). We further checked whether these nine modules were expressed during growth in FL media by growing each of the four isolates separately in FL media and comparing how their RNA expression patterns changed from early (G1) to the late (G2) growth phase (Fig. S12a). We found that eight out of the nine modules had a significant fold change of expression between G1 and G2, and these modules were considered as expressed in FL (Fig. S12b). Additionally, we selected compounds that were representative substrates of the eight expressed metabolic modules, and we tested if our isolates indeed could grow on these substrates (Supplementary Methods). Eventually, for each isolate, only modules that were expressed and growth-confirmed were considered as modules that were relevant for niche partitioning (Fig. 5b). The modules were related to several different categories of substrates, including amino acids and nucleotides as well as aromatic substrates. For example, the strain Cobetia10 from the late group expressed genes in the leucine and tyrosine degradation modules and could utilize the two compounds for growth. Since Neptunomonas4 did not have complete degradation modules and did not grow in these two substrates, these two amino acids may represent resources that are being partitioned in the late group. Similarly, Vibrio1 and its counterpart Psychromonas14 in the early group may be partitioning the amino acid tyrosine as well as the nucleobase pyrimidine. Meanwhile, Neptunomonas4 uniquely expressed the benzoate degradation pathway and catechol ortho-cleavage modules, two modules representing consecutive reaction steps in the degradation of aromatic compounds, and it was the only isolate capable of growing on sodium benzoate as the only carbon source. Thus, Neptunomonas4 likely was the sole utilizer of some of the aromatic compounds that were detected in FL (Fig. S1e).

Overall, our data suggest that the ability to better scavenge and break down nucleic acid monomers, amino acids, and aromatic compounds leads to low-level resource partitioning among the strains. Moreover, although polysaccharide degradation pathways were broadly shared, we observed differences in their expression along the growth trajectories that may further differentiate the strains (Fig. S12c). We caution that because of our strict filtering criteria, additional pathways may be involved in low-level resource partitioning. However, the pathways we detected cover most of the classes of compounds that are components of FL so that they are representative of a general pattern: although nearly all strains share mannitol as the major growth substrate, they are all differentiated along additional niches with substrates that are less abundant and of low concentration.

## Discussion

We show that, even in a highly dynamic system characterized by succession, functionally redundant bacteria can coexist due to resource partitioning of minor, less-abundant nutrients even though all organisms compete for a dominant substrate. Research to date has primarily focused on spatiotemporal niche partitioning to explain functional redundancy [12-17, 27, 33], and we also observed turnover of strains during phases of the succession. However, strains from within each phase and between different phases could stably coexist, and our analysis suggests that this was enabled by the differential use of substrates that were present at lower concentration. Stable coexistence of bacteria sharing a major substrate and partitioning minor substrates has also been demonstrated for the closely related *Lactobacillus* species from the bee gut [25], which is an overall stable system with limited substrate diversity. Our analysis extends this observation to the inherently unstable ocean system where diverse bacteria compete for many different substrates and is characterized by the rapid turnover on the time scale of hours to days [26].

The dynamics during the assembly of the initial and reconstituted communities match the observations of heterotrophic bacteria blooms that follow microalgal blooms in the coastal ocean. In these algal blooms, the division of labor for algal-derived substrate degradation is widely recognized to happen between the *Bacteroidetes* and *Alpha*, *Gammaproteobacteria*: *Gammaproteobacteria* have been shown to specialize on smaller organic compounds, while *Bacteroidetes* degrade the larger and more abundant biopolymers released by the algae [27, 34-36]. Our system differs in that we used the brown macroalgae *Fucus*, which is a stable member of the coastal community and leaches smaller molecules from dead biomass. In accordance with the inferred preference for smaller organic molecules, the majority of ASVs were *Gammaproteobacteria* (28 out of the top 40 ASVs in the succession), whereas only 1 *Bacteroidetes* was found in the top 40 ASVs. Our 10 selected isolates for functional redundancy analysis also had eight *Gammaproteobacteria* and only one *Bacteroidetes* (Arenibacter71). Consistent with the labor division in algal blooms, the only *Bacteroidetes* (Arenibacter71) isolate was also the “outlier” that showed a much lower functional redundancy with all other isolates, and it was also the only isolate that showed significant growth on the complex and recalcitrant polysaccharide *Fucoidan* found in FL (Supplementary Methods, Fig. S13). Moreover, we have shown previously that several *Vibrio* species are highly adapted to the colonization of *Fucus* biomass and degradation of the major cell wall polysaccharide alginate [37, 38], further underscoring that the observed dynamics may reflect natural processes.

Our work also demonstrates that certain species may have a tendency to consistently coassemble in successional dynamics. Although the relative influences of stochastic and deterministic processes between successional stages are a frequent topic of study [39], there has been little focus on the assembly process of bacteria that bloom simultaneously during one or more phases of the succession. These bacteria are often described by the function that represents their successional stage, such as pioneers for polysaccharide utilization [27, 32] or organisms capable of degrading recalcitrant organic carbon [40], which often represent their major redundant functions. Whether the observed coexistence of bacteria within an assembly stage is truly the effect of niche partitioning or is simply determined by their initial starting state remains largely unknown except when a clear syntrophic relationship is identified [41, 42]. The stable coexistence of isolates belonging to the same succession stage on *Fucus* leachate observed here signifies that their assembly during the succession is, at least, partially due to niche partitioning: although the inoculation ratio can strongly affect the relative abundances of the bacteria during short-term succession stages, the complete absence of a microbe from the succession stage is unlikely. Moreover, given that both the identity and relative abundance of coblooming microorganisms are often recurrent following phytoplankton blooms in nature [35, 43], it is likely that the resource-partition-based coassembly observed in these model consortia can be extended to natural communities and that some of these resource-partitioning strategies may have coevolved among community members.

Although our model consortium only had four bacteria, the fact that its equilibrium condition consists of two bacteria that are high, as well as two bacteria that are low in relative abundance, makes the community composition proportionally similar to typical species rank abundance distributions (RADs) in natural microbiomes. Typically, RADs are characterized by few dominant taxa and a long tail of “rare” taxa. Similarly, functional RADs in microbiomes are often characterized by few dominant genes and a long tail of “rare” genes [7]. Species-function incidence matrices of human gut microbiomes further show that such RADs on both the taxonomic and functional levels are a consequence of few central metabolic and growth functions being shared among many different taxa and large numbers of specialized functions being present in only a few taxa [44, 45]. This means that in the niche space, only a small number of niches will be “crowded,” while most might be quite “empty,” i.e. a few common substrates will be subject to heavy competition by many taxa, while other rarer substrates will be utilized by one or a few more specialized taxa. We expect that this concept is suitable for microorganisms in various environments because dominant and minor resources exist nearly everywhere. In the coastal ocean, mannitol may be the major resource for competition in “sweet spots” surrounding brown algae [46, 47], whereas for seagrasses, this resource may be sucrose [48], and for spring algal blooms, the resource competed for in the early “die off” phase is often considered to be laminarin [27]. We speculate that the simultaneous competition for a major resource, while partitioning other minor resources, may be especially common in environments with strong fluctuation and pulses of substrates, and particularly when there is a substantial release of a singular type of carbon source or carbohydrate. Such scenarios are not only limited to the coastal ocean but can also occur in the plant rhizosphere [49] and dryland soils [50] as well as in the mammalian gut [51]. Therefore, low-level resource partitioning could be common in natural microbiomes and may be one of the major drivers of microbial coexistence in the wild.

## Methods

### Media preparation and chemical analysis of *F. vesiculosus*

FL media was prepared in batches from dissolving sun-dried and grounded *F. vesiculosus* in a modified version of the Tibbles-Rawlings minimal media [33, 52]. The *Fucus* was collected from the rocky shorelines of Canoe Beach (see below for exact coordinates) on 12 July 2014. The concentration of dissolved organic material in FL media is estimated to be ~0.022% (w/v). Detailed methods for preparing the FL media and determining its chemical composition are described in the Supplementary Methods.

### Sampling and incubation

Coastal ocean seawater samples and shallow marine sediment samples were both collected from Nahant, MA, USA on 5 November 2014. The sampling sites for the seawater (Canoe Beach, 42° 25′ 10.0” N, 70° 54′ 25.4’ W) and sediment samples (42° 25′ 44.4” N, 70° 55′ 48.0” W) were ~2 km apart. Both the seawater and sediment samples were filtered onto 0.22-μm Sterivex filters (Millipore, SVGP01050) using a peristaltic pump, and the filter papers were cut and used for DNA extraction and inoculation (Supplementary Methods). Filter pieces used for inoculation were placed in triplicate microcosms that were pump-aerated 4-l flasks containing 3 l of FL media. The flasks were continuously stirred at 150 rpm at room temperature for 9 days, with samples taken at various points across the time period (Supplementary Methods).

### Spent-media experiments

Spent-media experiments were performed in 10 ASV × 10 ASV and 4 ASV × 4 ASV combinations (Supplementary Methods). The fraction of growth of one ASV in the spent-media of another was calculated as ratio of the maximum cell density reached in the spent-media to the maximum cell count reached in “unspent” FL media. The functional overlap between two ASVs (i.e. *A* and *B*) is thus calculated as $1-\mathrm{Cell}\ \mathrm{count}\ \left(A\ \mathrm{in}\ \mathrm{spent}\ \mathrm{media}\ \mathrm{of}\ B\right)/\mathrm{Cell}\ \mathrm{count}\ \left(A\ \mathrm{in}\ \mathrm{FL}\ \mathrm{media}\right)$.

### Individual growth curves

Isolate precultures were prepared in triplicates as described in the Supplementary Methods and were then grown in 48-well deep-well blocks and by shaking at 300 rpm on a tabletop shaker at room temperature. Samples were taken at 15–20 time points covering all growth phases of the isolate according to the standard protocol for measuring biomass accumulation via FACS. Maximum growth rates during the early growth phase (0–12 h) for each growth curve was acquired by fitting segments of linear models to the log-transformed cell concentrations and by selecting the segment with the maximum growth rate (slope) by using the function fit_easylinear (quota = 1) in the R package growth rates [53]. Segments included five consecutive time points if the fit quality for the maximum growth rate segment was high (*R*^2^ > 0.9) and otherwise four consecutive time points.

### Competition experiments

Isolate precultures for all competition experiments were prepared according to the standard protocol (Supplementary Methods). For all the pairwise competitions, the isolate cultures were mixed at 10:90 or 90:10 to mimic invasion of one isolate by another. For 4-way competitions, a total of 12 combinations were tested to make sure that we had high, medium, low starting percentages for all isolates (Table S1).

During the competition experiment, the communities were grown in 96-well blocks at room temperature containing 300 μl media in each well while shaking on a tabletop shaker at 300 rpm (except Cycle 1, see “Succession of 4 strain model communities” in Supplementary Methods), for nine growth-dilution cycles. Community composition at the end of Cycles 1, 5, 8, and 9 were determined at the end of these cycles under the standard protocol (Supplementary Methods).

### Whole-genome sequencing and functional annotation

Genomes of the 10 isolates representing the 10 selected ASVs for the model community were obtained (Supplementary Methods) and the quality of each genome was checked via checkM v1.0.7 [54]. All genomes were >98.5% complete with <5% contamination. The distribution of genes across KEGG pathway modules (Supplementary Methods) in each organism were used to calculate the functional distance (FD) between the organisms (FD(*A*, *B*) = 1 − cor(module_distribution(*A*), module_distribution(*B*)), Pearson’s correlation). Module completeness is calculated as the average completeness of all reactions included in the module (completeness of each reaction = #of reaction specific KOs found in genome/total # of KOs in reaction).

###  RNA sequencing of selected isolates

Samples for transcriptomics were taken before and after the diauxic shifts for Psychromonas14 and Vibrio1 and in early and late log phase for Cobetia10 and Neptunomonas4 (Supplementary Methods). After RNA extraction (Supplementary Methods), sequencing libraries were prepared with the KAPA RNA HyperPrep Kit and sequenced on an HiSeq 2000 System (Illumina) for single-end 50-bp reads at the BioMicro Center (Massachusetts Institute of Technology, Cambridge, MA).

### Gene expression quantification and differential expression analysis

Transcript quantification from the RNA-seq data was performed by competitively mapping reads in each sample to annotated genes from all four isolates Psychromonas14, Vibrio1, Cobetia10, and Neptunomonas4 as reference. The reference genes were indexed with Salmon 0.14.1 [55](–numBootstraps 200), and read quantification was performed in quasi-mapping-based mode (quant –seqBias –gcBias –useVBOpt –validateMappings –numBootstraps 200). Differential gene expression between G1 and G2 growth phase for each isolate was analyzed using the R package DESeq2 [56] to output the log2-fold expression change (and adjusted *P*-value) of each gene, with genes with <10 counts in total being omitted from the analysis. Genes are assigned to KEGG modules as well as CAZy families according to the previous functional annotation of each isolate genome. For each genome, KEGG modules with equal or less than one-third of functional genes assigned were considered to be incomplete, and KEGG modules with equal or more than two-thirds of functional genes assigned were considered to be complete. The integrated log-fold change of each complete KEGG module (or CAZy family) was calculated as the sum of log-fold expression changes for all genes in the module, and the *P*-values were combined according to the Simes’ method [57] for obtaining the integrated *P*-value for gene expression change of the module (or CAZy family).

## Supplementary Material

TableS1_wrad013

TableS2_wrad013

seaweed_succession_sup_figures_clean_final_wrad013_1

seaweed_succession_sup_methods_clean_wrad013_1

## Data Availability

All relevant amplicon, genomic, and transcriptomic data will be released under BioProject PRJNA905473, and all codes for data processing will be uploaded to https://github.com/cusoiv.
