# Supplementary material for: Low-level resource partitioning supports coexistence among functionally redundant bacteria during successional dynamics
Source: ISME J. 2024 Jan 10;18(1):wrad013. doi: 10.1093/ismejo/wrad013 (PMC10811730; doi:10.1093/ismejo/wrad013)
Supplement: seaweed_succession_sup_figures_clean_final_wrad013_1 [file seaweed_succession_sup_figures_clean_final_wrad013_1.pdf]

Figure S1

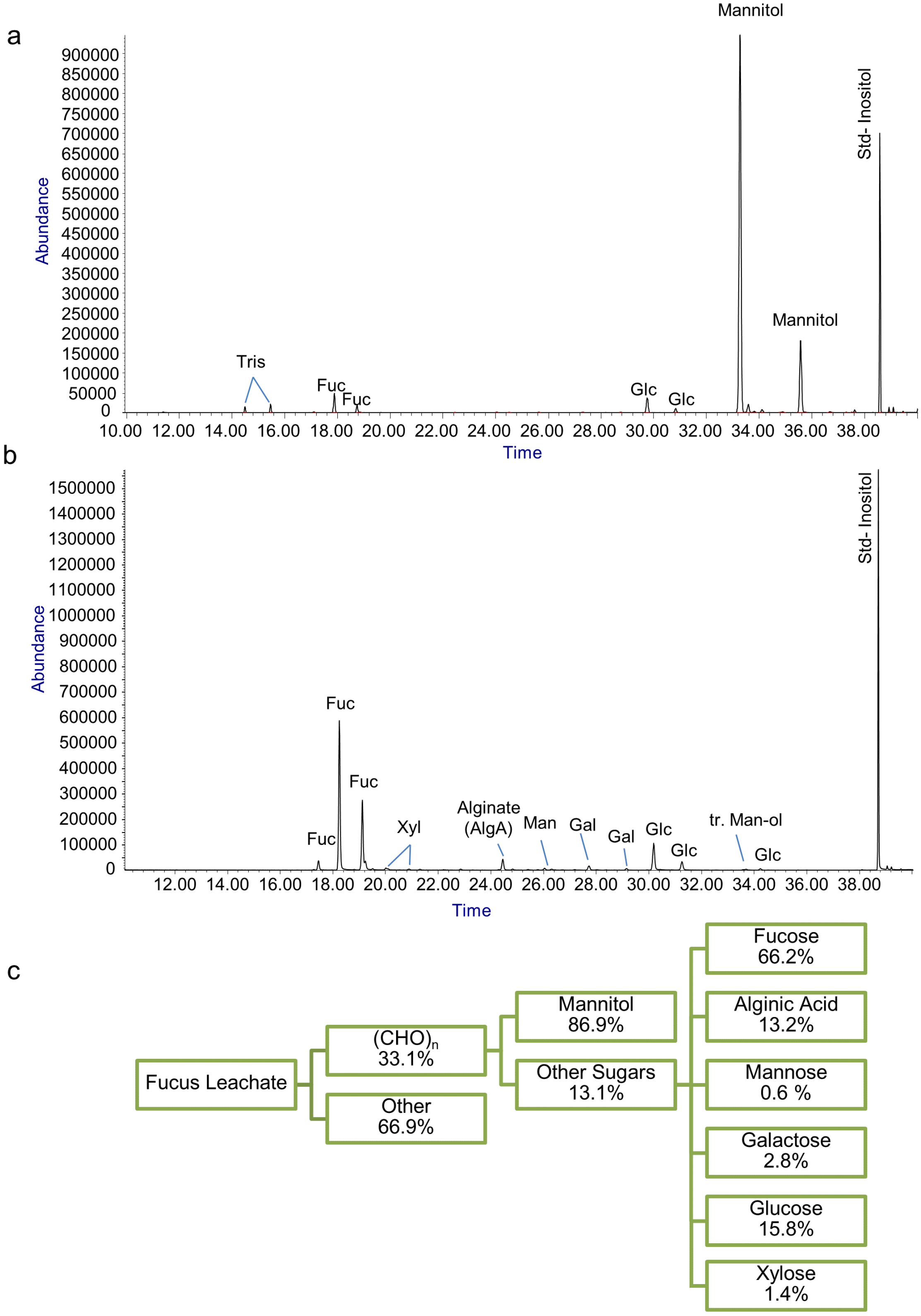
a
Mannitol
Std- Inositol
Mannitol
Abundance
900000
850000
800000
750000
700000
650000
600000
550000
500000
450000
400000
350000
300000
250000
200000
150000
100000
50000
0
Tris
Fuc
Fuc
Glc
Glc
10.00 12.00 14.00 16.00 18.00 20.00 22.00 24.00 26.00 28.00 30.00 32.00 34.00 36.00 38.00
Time
b
Std- Inositol
Abundance
1500000
1400000
1300000
1200000
1100000
1000000
900000
800000
700000
600000
500000
400000
300000
200000
100000
0
Fuc
Fuc
Fuc
Xyl
Alginate
(AlgA)
Man
Gal
Gal
Glc
Glc
tr. Man-ol
Glc
12.00 14.00 16.00 18.00 20.00 22.00 24.00 26.00 28.00 30.00 32.00 34.00 36.00 38.00
Time
c
Fucus Leachate
$(CHO)_n$ 33.1%
Other 66.9%
Mannitol 86.9%
Other Sugars 13.1%
Fucose 66.2%
Alginic Acid 13.2%
Mannose 0.6 %
Galactose 2.8%
Glucose 15.8%
Xylose 1.4%

d

Glycosyl residue	Seaweed glycosyl linkage composition after removal of mannitol		
	Sulfated*	De-sulfated*	Change (%)
3-Fuc*p*	0.0	3.0	3.0
4-Fuc*p*	0.0	8.4	8.4
3,4-Fuc*p*	26.3	62.3	36.0
2,3-Fuc*p*	11.7	7.0	-4.7
2,3,4-Fuc*p*	41.1	12.0	-29.1
2,3-Man*p*	2.5	1.0	-1.5
2,3/2,4-Gal*p*	6.6	2.2	-4.4
2,3-Glc*p*	1.9	0.9	-1.0
2,3,4-Gal*p*	3.3	0.6	-2.7
3,4,6-Man*p*	2.2	2.1	-0.1
3,4,6-Glc*p*	2.9	0.4	-2.5
2,3,6-Gal*p*	1.4	0.2	-1.2

*, (%) the values represent the relative percent calculated from EI (electron impact) detector area response

e

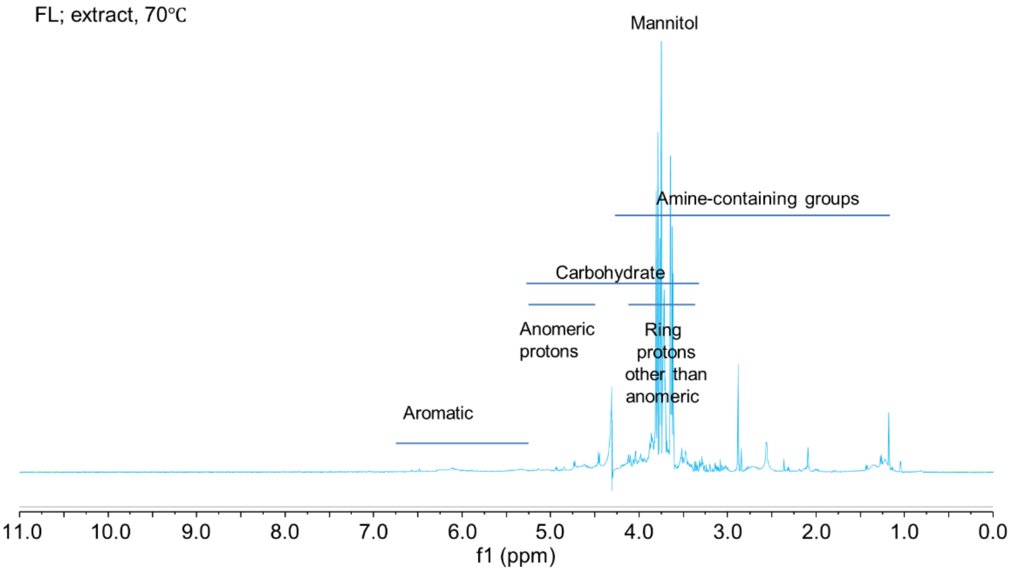

Figure S1 Carbohydrate constituents and chemical groups in *Fucus* Leachate (FL). GC-MS chromatogram of glycosyl residues in the *Fucus* leachate **(a)** before and **(b)** after mannitol was removed by dialysis (1000 Da cutoff). **(c)** Distribution of glycosyl components in *Fucus* leachate and their relative percentages. **(d)** Glycosyl linkage analysis of the mannitol-deprived portion of the *Fucus* leachate **(e)** H^1-NMR analysis of FL. In addition to chemical shifts due to mannitol and other carbohydrates, the peaks in the 1-5 ppm region suggest the existence of amine-containing groups (e.g. from protein), while the signals at approximately 6ppm indicate the presence of aromatic compounds.

Figure S2

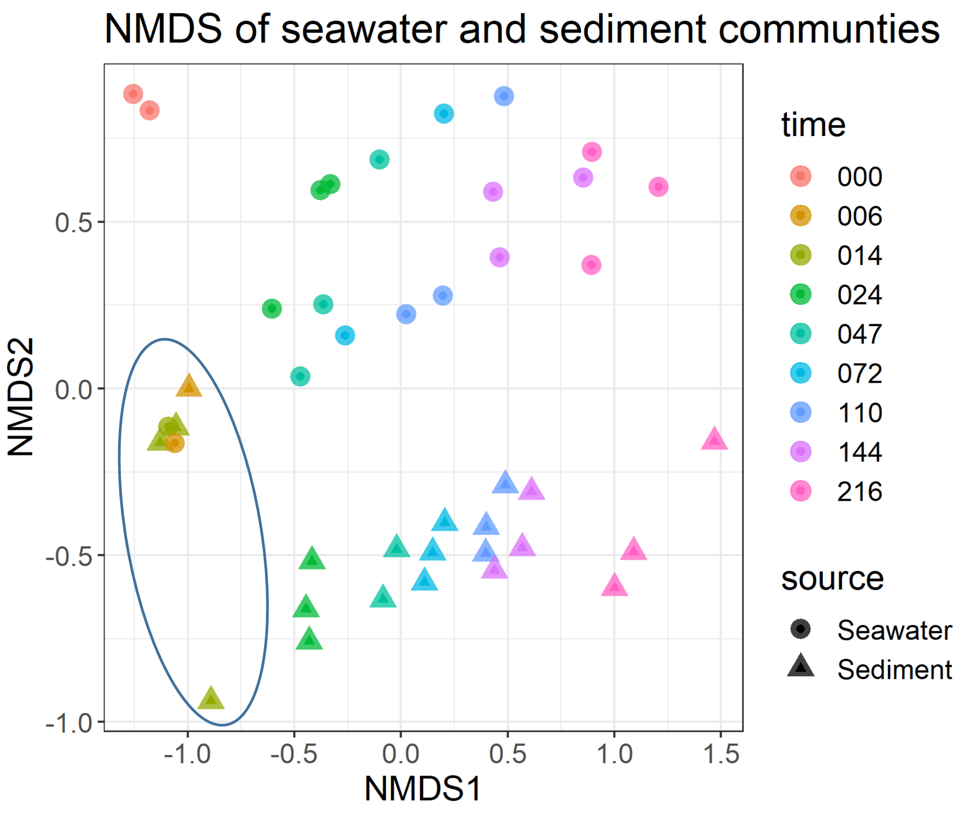

Figure S2 Seawater and sediment inoculated communities are more similar during early time points in the succession. Non metric multidimensional scaling (NMDS) of the compositions of seawater and sediment inoculated communities evaluated by 16S rRNA gene amplicon sequencing. Points in the oval represent communities from early (6 h, 14 h) time points.

Figure S3

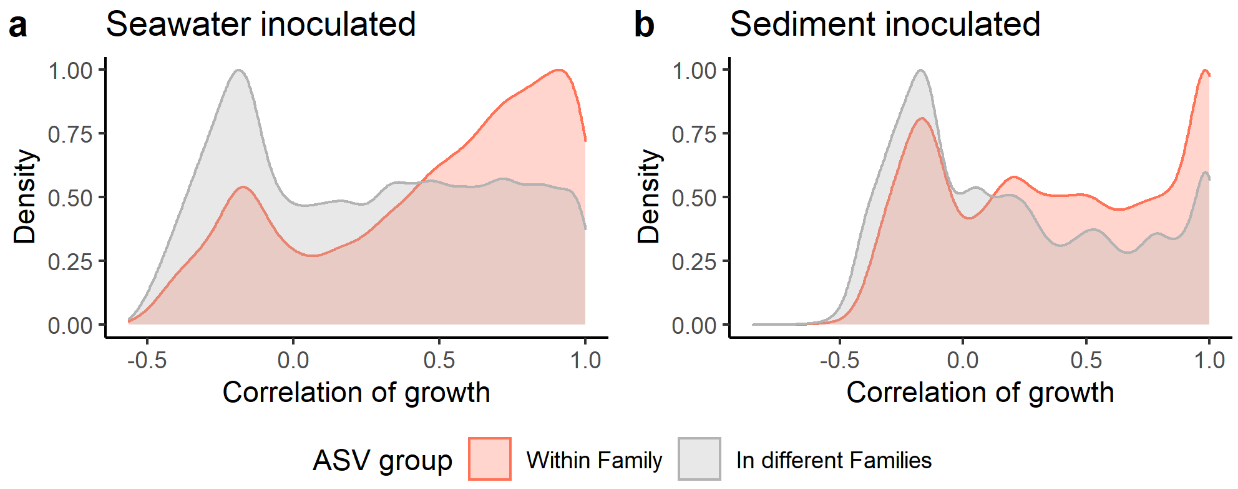

Figure S3 Succession patterns are more similar for ASVs within the same family in the two earliest communities. Distribution of the Pearson correlations between absolute abundance trajectories for any two ASVs within the same family [red, n=11196(seawater), 8351(sediment)] and in different families [gray, n=71748(seawater), 56165 (sediment)] for (a) seawater and (b) sediment inoculated communities. Only ASVs that grew to more than 10^3 cells/mL were selected for the analysis.

Figure S4

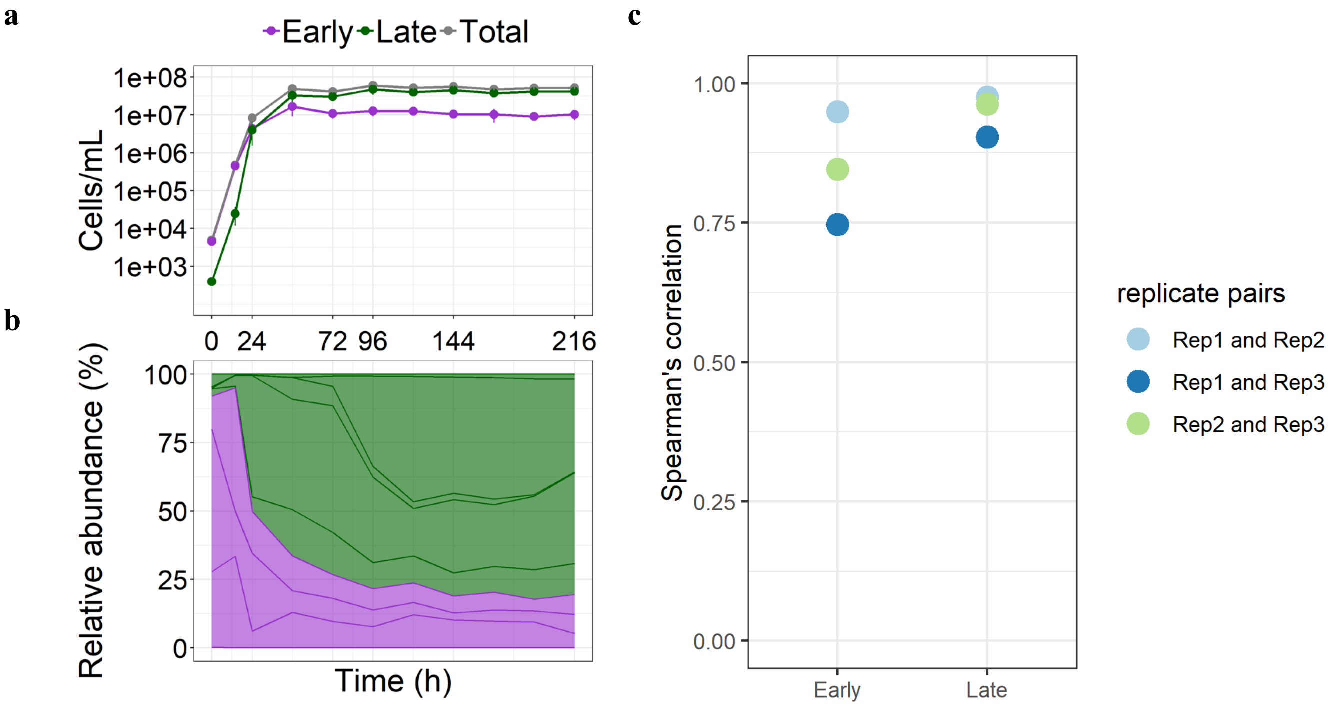

Figure S4 Isolate-constituted communities consistently recapitulate dynamics in natural communities (a) Growth profile in absolute cell counts of the synthetic 10 isolate community, grouped by early and late isolates. Cell counts in the system did not decline after 48h as in the natural communities. Error bars are standard deviations (n=3). (b) Community composition, shown as the average (n=3) relative abundances over time of the reconstructed 10-isolate community. Early isolates are shown in purple, while late isolates are shown in green. Community growth before 24 h was majorly driven by bacteria in the early group, and after 24h, the late bacteria expanded to approximately 75% of the community. (c) Spearman's correlation for the absolute abundance of all isolates belonging to the early or late assembly group between time points 14-144 h for the 10-isolate communities across all replicate pairs.

Figure S5

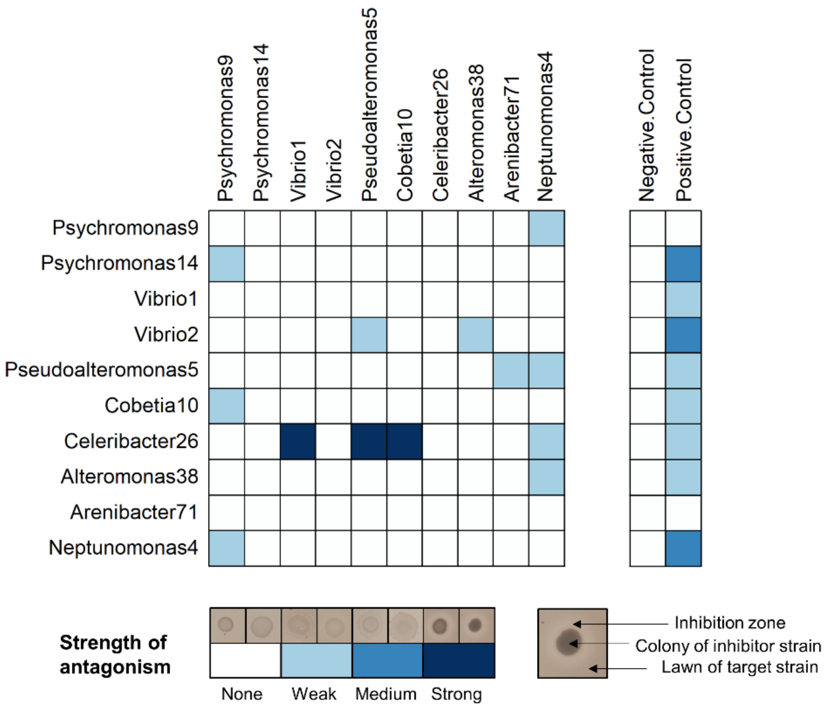

Figure S5 Antagonism between selected isolates are rare and weak. Summary of the Burkholder diffusion assay results between the 10 selected isolates. For the 10×10 matrix, the color of each square represents the extent of antagonism between the potential inhibitory strain (columns) and the target strain (rows). The strength of antagonism is measured as the clear areas (size + opacity) around the potential inhibitory strain growing on the target lawn (right bottom panel), and examples of no, weak, medium and strong antagonism are illustrated in the left bottom panel. The 10×2 matrix on the right represents all positive controls [potential inhibitory strain is a known “killer” Vibrio (column) against all target strains (rows)], and all negative controls [blank media (column) against all target strains (rows)].

Figure S6

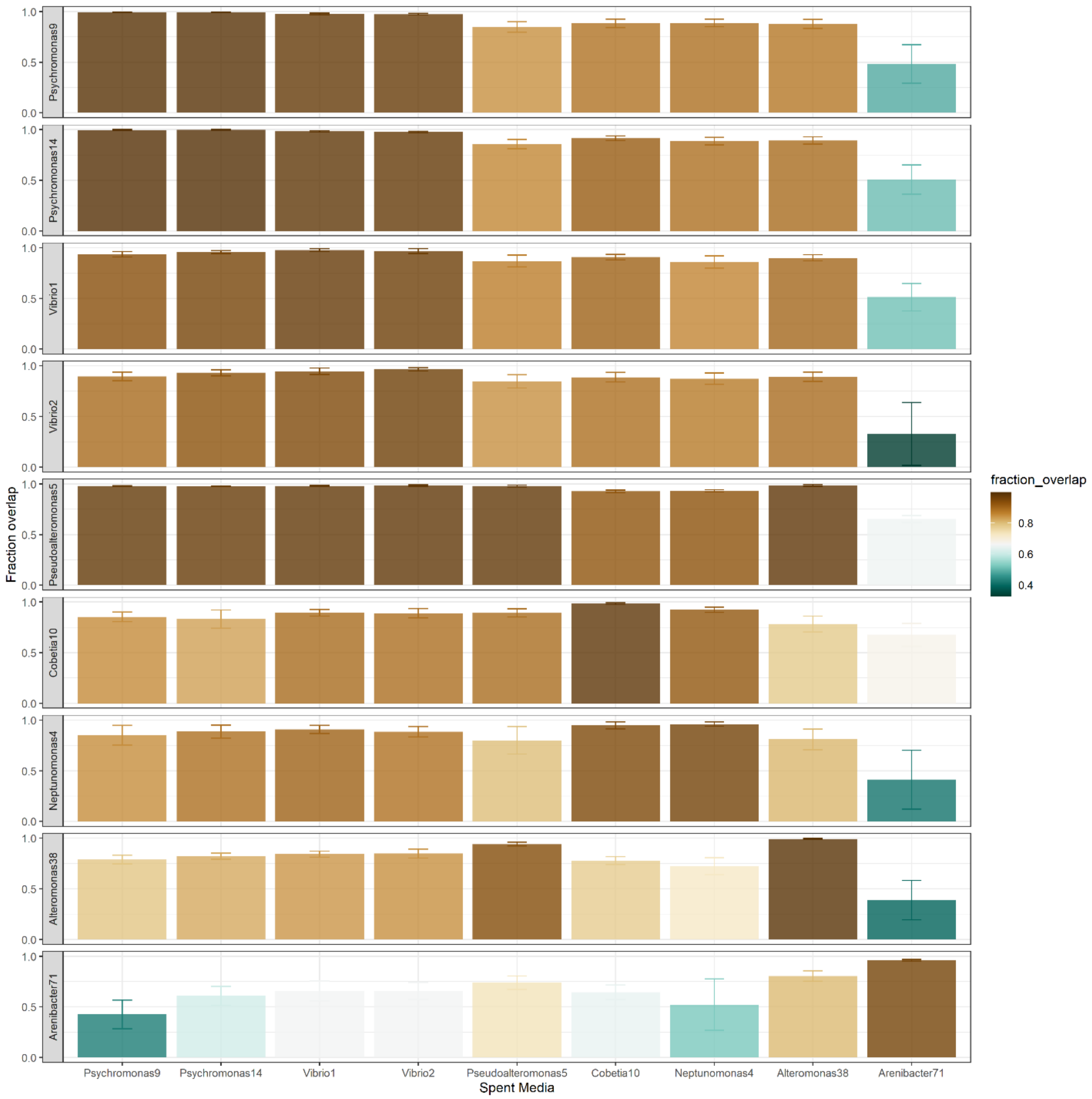

Figure S6 Functional redundancy is widespread among isolates of the model community. Proportional growth of each isolate (rows) in the spent media of another isolate (columns). Filled regions represent overlap of resource use between two isolates as evidenced by reduced growth compared to growth in fresh FL media. Data as in Fig. 2a except that it is in a format that allows visualization of the standard deviations of three replicates.

Figure S7

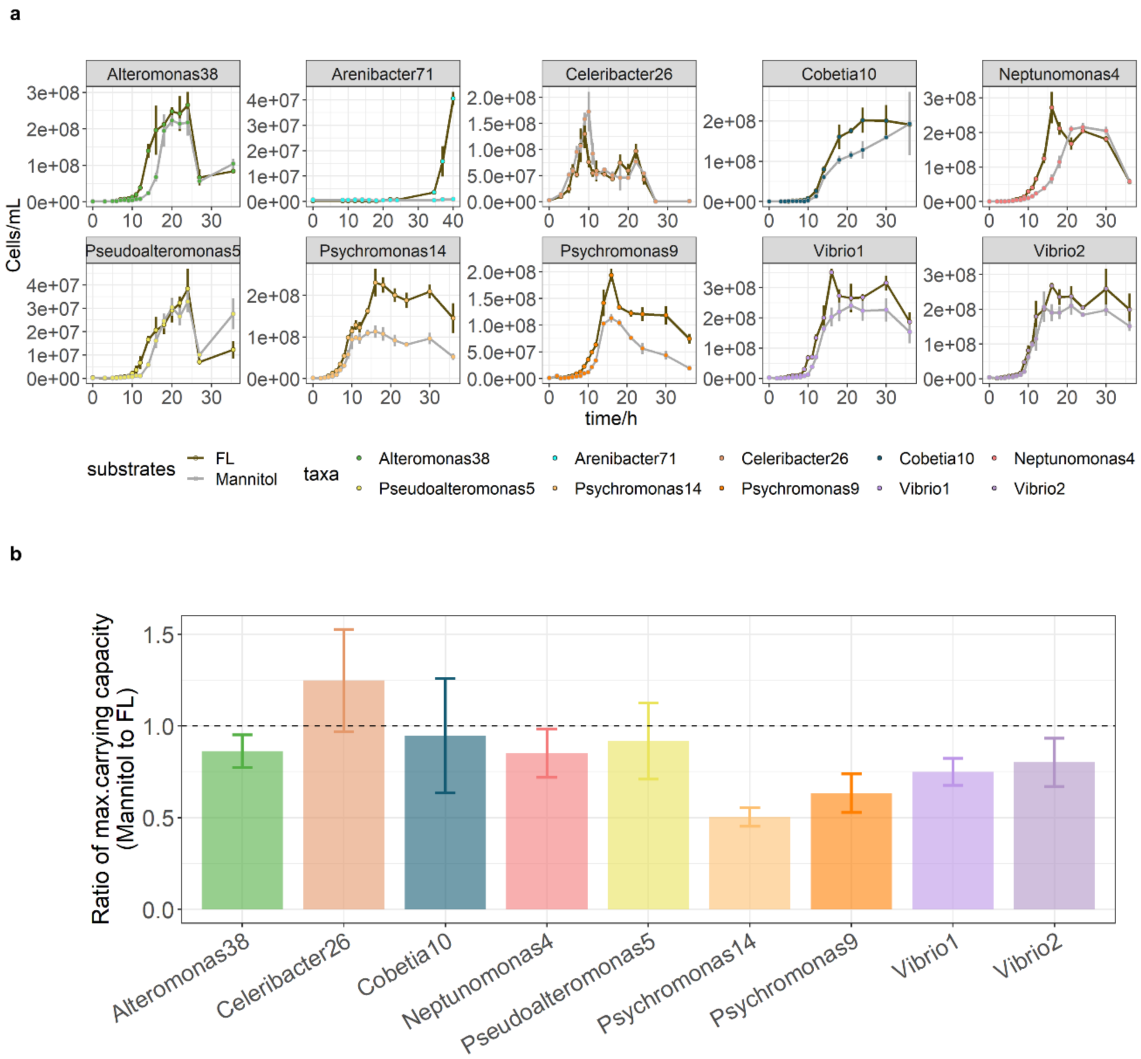

Figure S7 Comparison of individual growth behavior of all selected isolates in FL and 0.0047% mannitol media. (a) Curves in dark green represent growth in FL, while curves in grey represent growth in 0.0047% mannitol. Error bars are standard deviation (n=3). (b) Ratio of maximum carrying capacity (maximum cell count reached within 36h of growth) for every ASV grown in mannitol vs. FL.

Figure S8

a

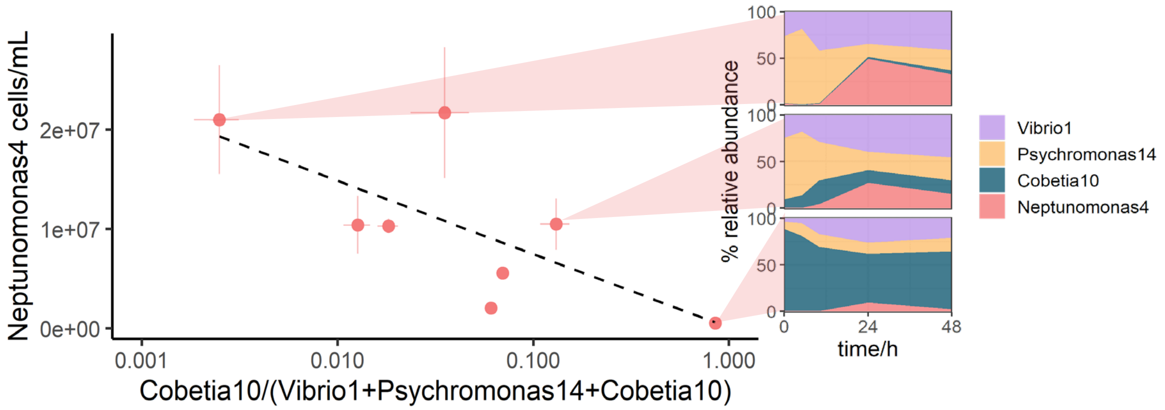

b

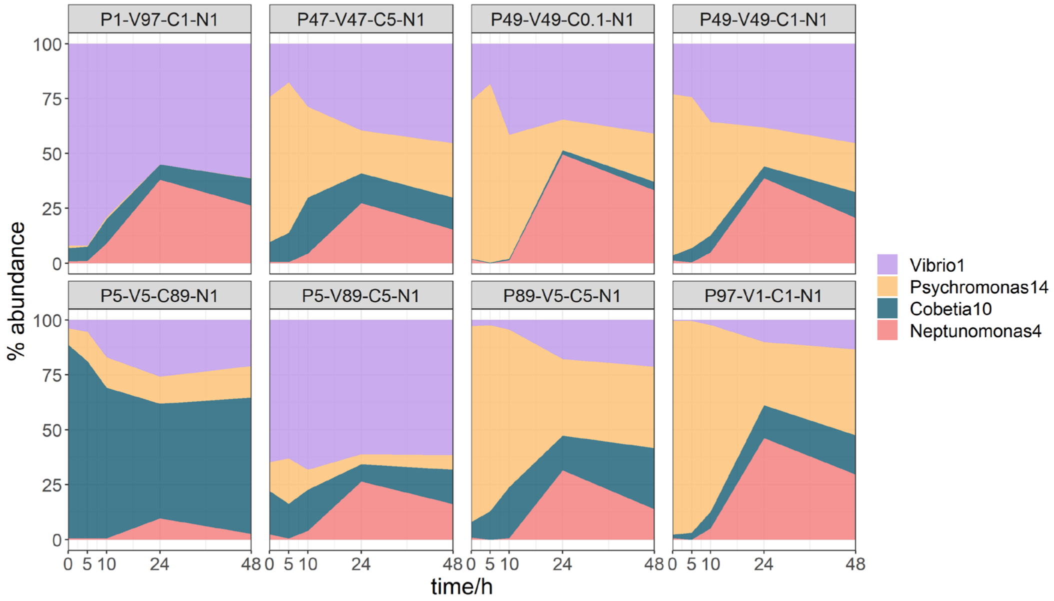

Figure S8 Evaluation of founder effects in short-term succession dynamics of the 4 strain model communities. a Left: Relationship between the average cell density (n=3) of Neptunomonas4 at 48 h and the relative abundance of Cobetia10 at inoculation (t=0 h). Right: Examples of short term (within 48 h) succession patterns that represent conditions where Cobetia10 are at high, medium and low starting concentrations, Vibrio1 and Psychromonas14 have equal starting concentrations, and Neptunomonas4 is at 1% in the inoculum. **b** Community composition changes for all 8 Vibrio1, Psychromonas14, Cobetia10 and Neptunomonas4 combinations (for all the points in **a**) over 48 h. The title of each facet represents the varying inocula where the letter denotes the strain (P=Psychromonas14, V=Vibrio1, C=Cobetia10, N=Neptunomonas4) and the numbers the intended percentages in the inoculum.

Figure S9

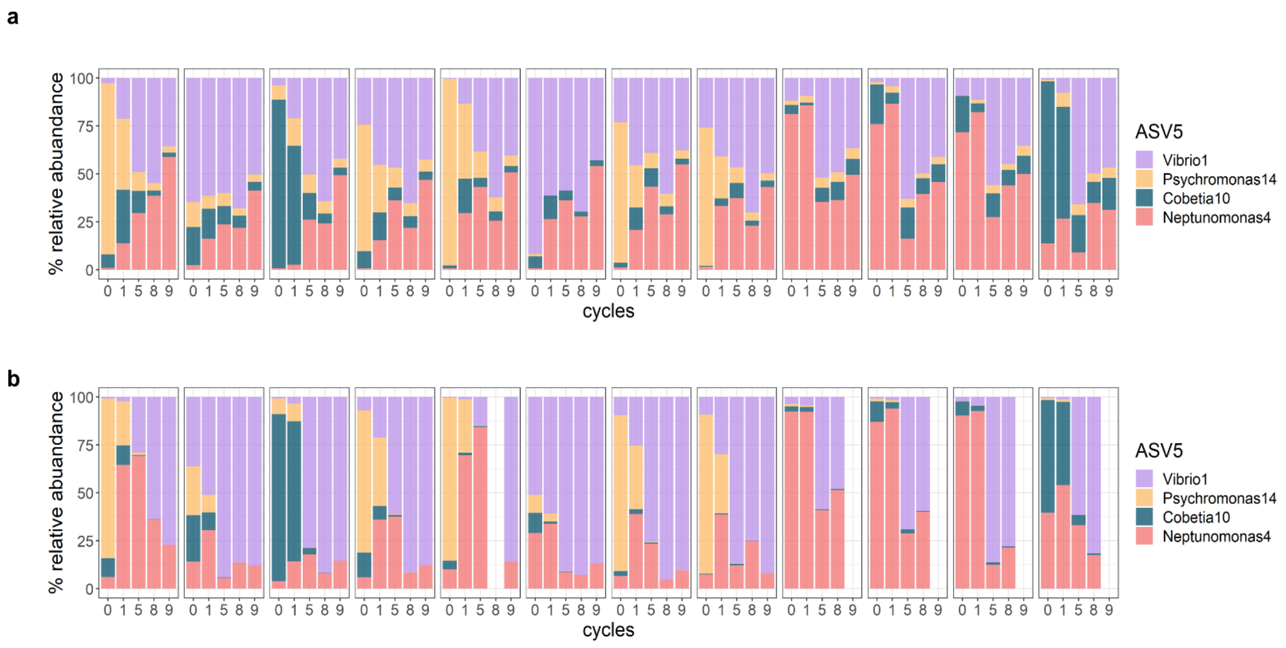

Figure S9 Community composition changes in 4-way competition experiments. Community composition changes over 9 growth-dilution cycles for all 12 sets of 4-isolate communities in (a) FL and (b) 0.0047% mannitol media.

Figure S10

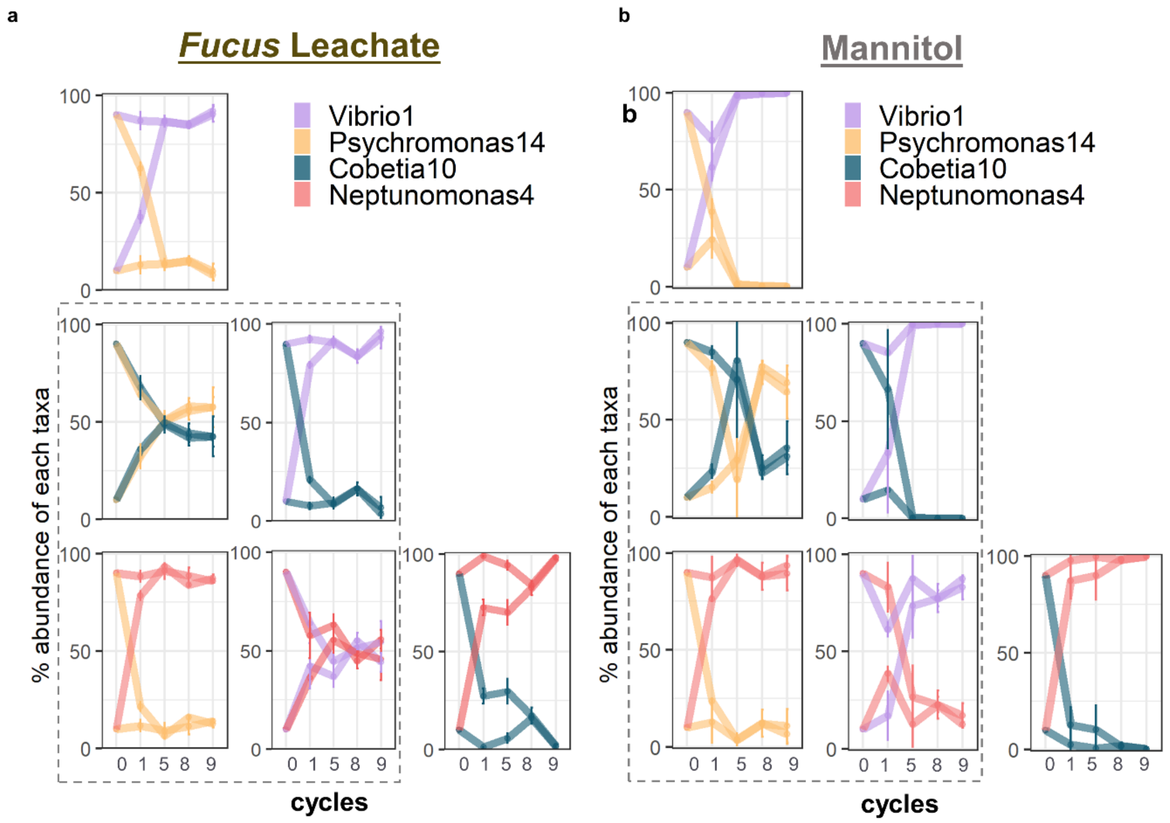

Figure S10 Trajectories for pairwise competition experiments

Changes in community composition for all pairwise competitions over 9 growth-dilution cycles in (a) FL media and (b) 0.0047% mannitol media. Error bars represent standard deviation (n=3). Graphs in the grey dashed box represent communities containing isolates from different assembly groups.

Figure S11

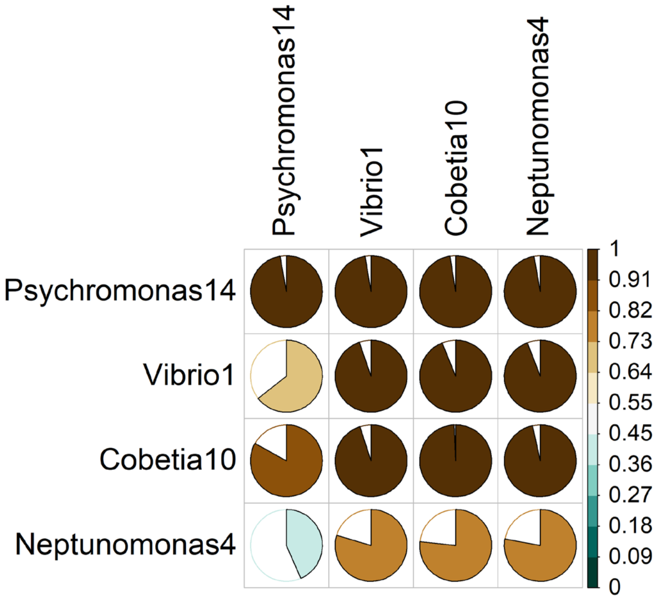

Figure S11 Potential cross-feeding between selected isolates.

Proportional growth of each isolate (represented in rows) in the spent media of another isolate after 48h of growth in 0.0047% mannitol (represented in columns). Since mannitol is utilized completely by all 4 isolates within 48h, any growth (white areas) is likely the result of cross-feeding on metabolic byproducts.

Figure S12

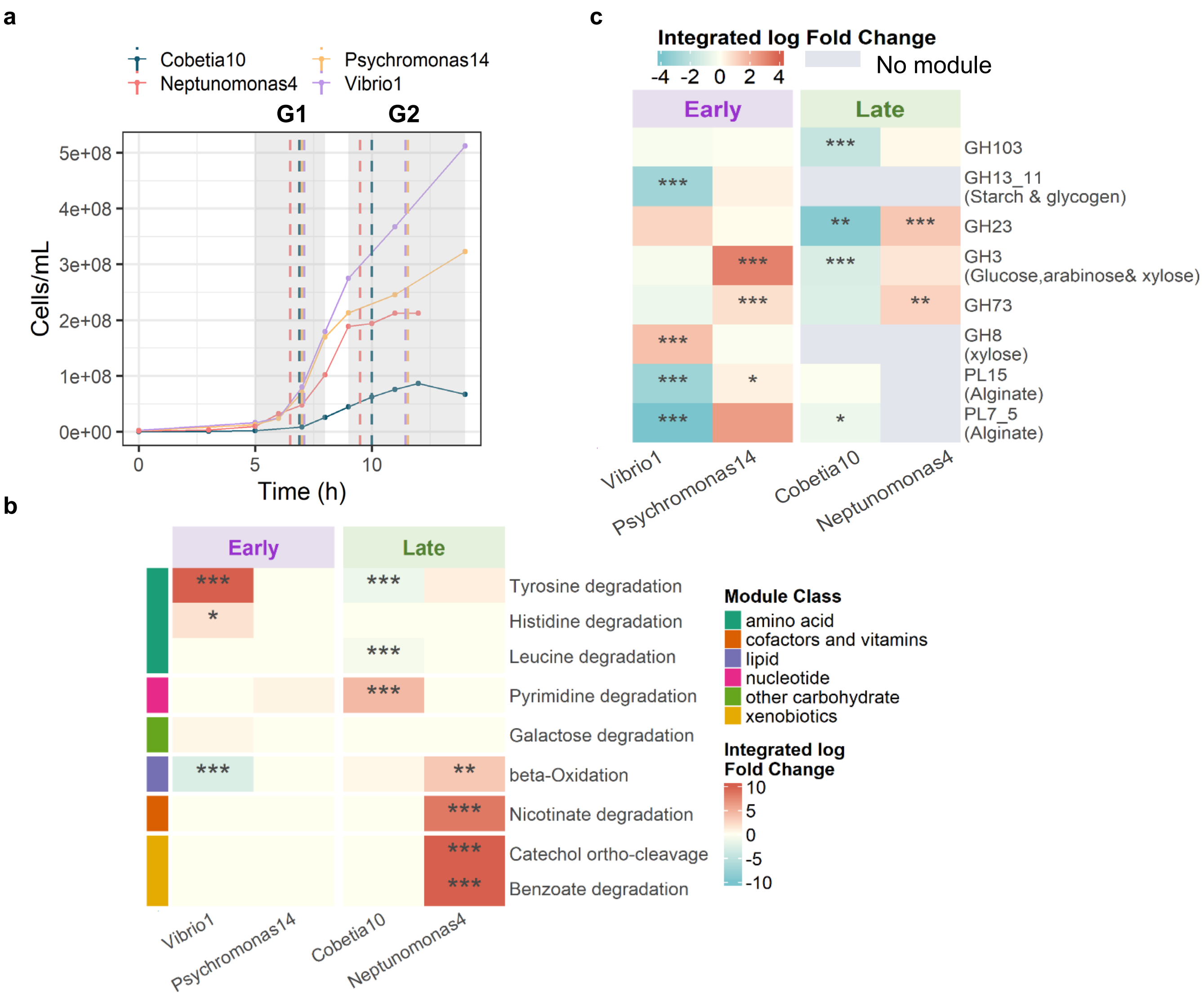

Figure S12 KEGG modules are differentiated among isolates on the expression level.

a Average growth curve (n=3) and sampling times for RNA-seq analysis for all 4-isolates growing individually in FL media. Vertical dotted lines represent the sampling times for each isolate, with samples taken in the G1 growth phase representing growth before the diauxic shift/early logarithmic growth, and G2 representing growth after the diauxic shift/ late logarithmic growth. Samples that were taken at exactly the same time were jittered slightly for visualization. **b** Heatmap of log2 fold module expression change between G1 and G2 growth phase for all KEGG modules that had different completeness for isolates within at least one assembly group, as well as different expression levels between G1 and G2 for the complete module. The p-values for each module are combined p-values of all genes in the module with the Simes' method. ***, p<0.001, **, p<0.01, *, p<0.1. **c** Heatmap of log2 fold module expression change between G1 and G2 growth phase for all CAZy families that were either unique to one isolate within at least one assembly group, or are differently up- or downregulated between the two growth phases. The p values for each CAZy family are combined p values with the Simes'method of all genes annotated as belonging to the family. ***, p<0.001, **, p<0.01, *, p<0.1.

Figure S13

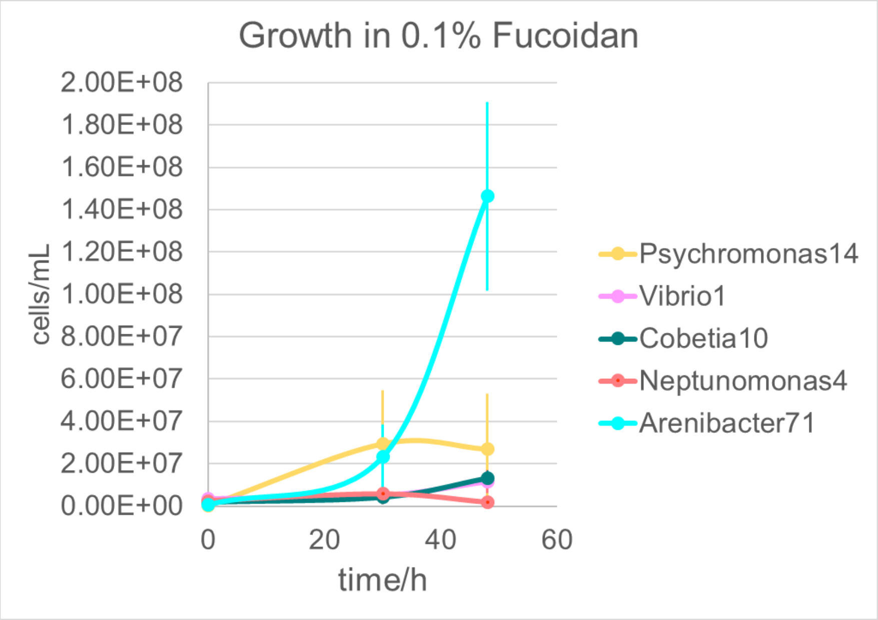

Figure S13 Growth of selected ASVs in 0.1% Fucoidan

The Fucoidan used was >95% pure and thus likely contained a small amount of other seaweed sugars. Therefore, we only consider growth to at least 10^8 cells/mL as positive growth on Fucoidan.

Figure S14

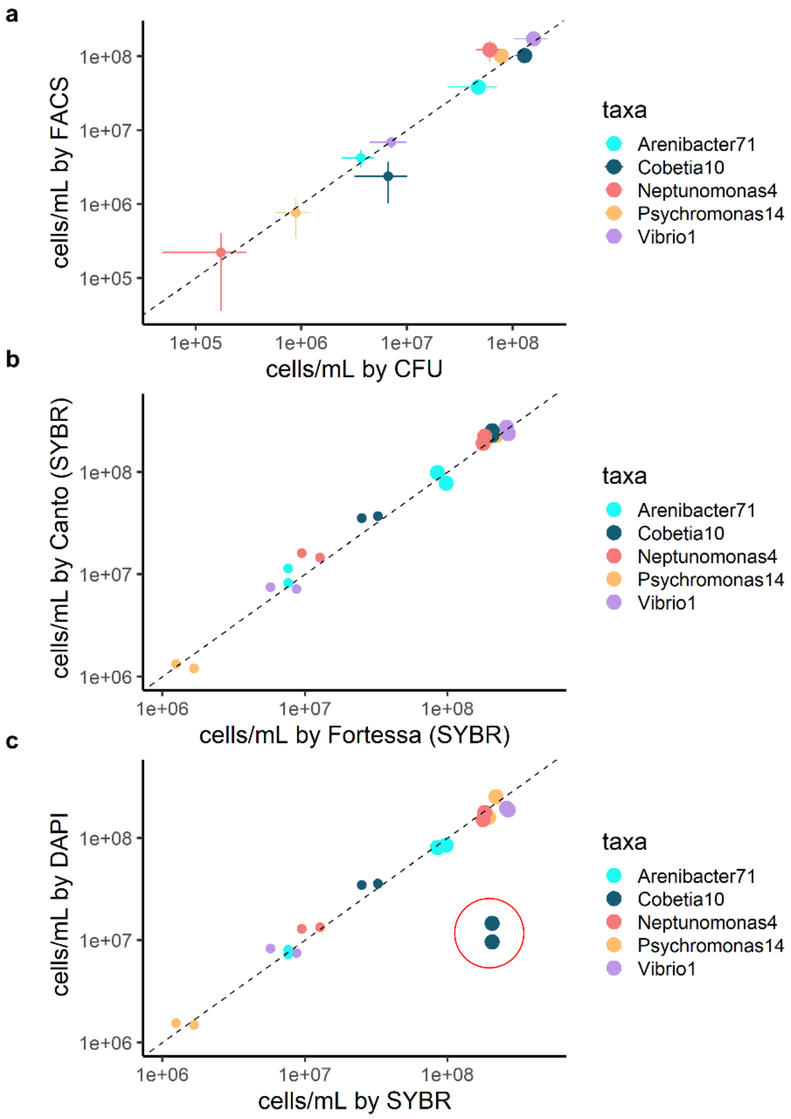

Figure S14 Validation of Flow cytometry methods

a) Comparison of FACS cell counts to CFU for 5 ASVs in early log (smaller points) and early stationary phase (larger points). Error bars represent standard deviation from three biological replicates. Black dotted line represent CFU counts=FACS cell counts.
b) Comparison of FACS cell counts on the BD Fortessa machine to BD Canto machine for 5 ASVs in early log (smaller points) and early stationary phase (larger points). Black dotted line represent that the cell counts from the two machines are equal.
c) Comparison of FACS cell counts on the BD Fortessa machine with DAPI vs. SYBR stain for 5 ASVs in early log (smaller points) and early stationary phase (larger points). Black dotted line represent that the cell counts from the two stains are equal. Red circle represents Cobetia10 at stationary phase, which did not stain well with DAPI.

Figure S15

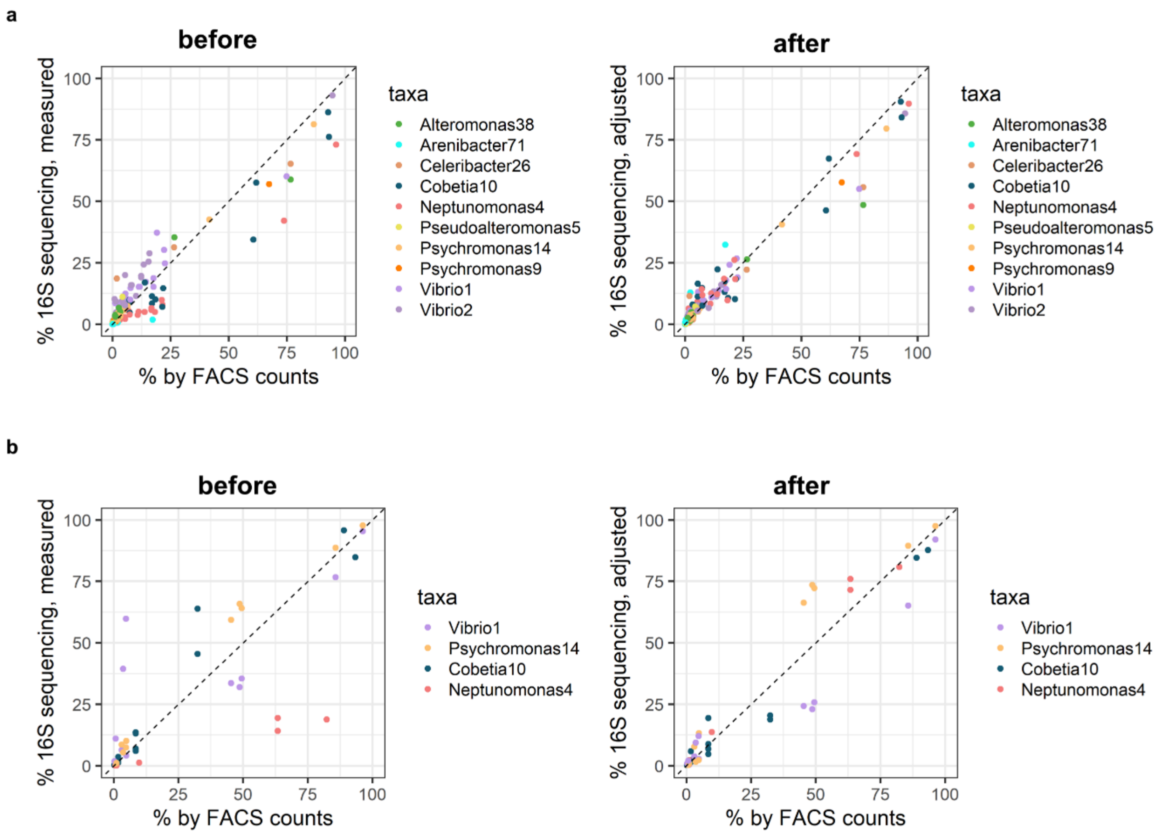

Figure S15 Correction for sequencing related bias for synthetic communities. Comparisons of the observed proportions (by 16S rRNA amplicon sequencing) of individual ASVs to the actual proportions (by mixing specific volumes of monocultures with known cell density), before and after correction for estimated bias (methods), in a) the 10 ASV synthetic community and b) the 4-ASV synthetic communities. Points in figures represent the average of 2 (a) or 3 (b) replicates. Diagonal lines represent observed proportions equal actual proportions.

Figure S16

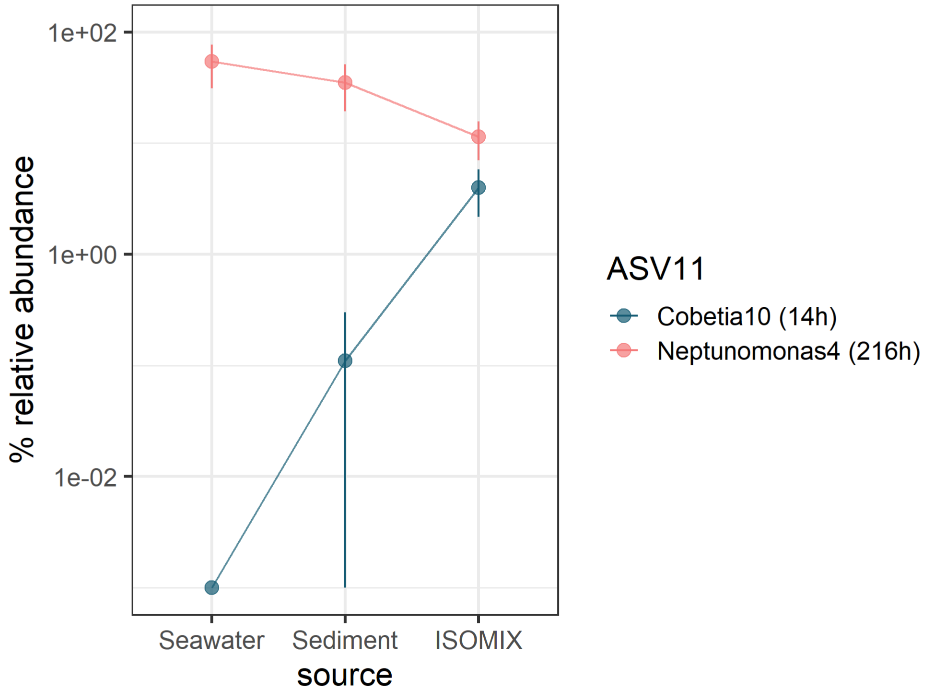

Figure S16 General trend for Cobetia10 and Neptunomonas4 in the natural and isolate inoculated communities. Relative abundances of Cobetia10 (14h, early time point) and Neptunomonas4 (216h, late time point) for three differently inoculated communities. Error bars are standard deviation (n=3).

Figure S17

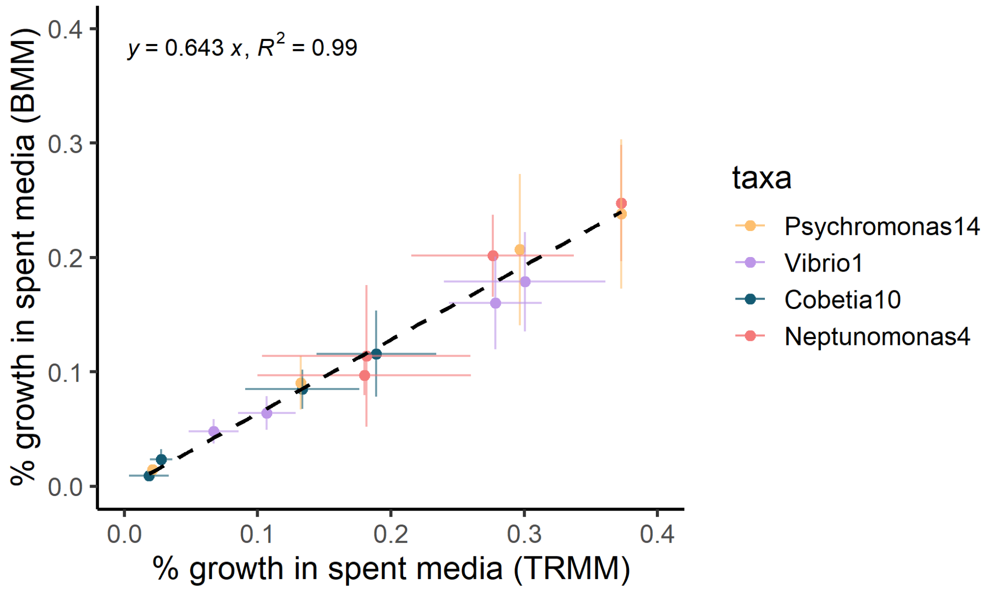

Figure S17 Comparison of niche overlap patterns of the four ASVs in bicarbonate based FL media (BMM) to Tris based FL media (TRMM). Each colored dot represents the portion growth of an ASV isolate in the spent media of another ASV in either TRMM or BMM. Black dotted line represents the linear fit of the proportional growth in BMM vs. TRMM. Error bar represents standard deviation of three biological replicates.
