# Supplementary material for: Low-level resource partitioning supports coexistence among functionally redundant bacteria during successional dynamics
Source: ISME J. 2024 Jan 10;18(1):wrad013. doi: 10.1093/ismejo/wrad013 (PMC10811730; doi:10.1093/ismejo/wrad013)
Supplement: seaweed_succession_sup_methods_clean_wrad013_1 [file seaweed_succession_sup_methods_clean_wrad013_1.pdf]

Supplementary Methods

FL media preparation

The *Fucus* collected was washed, sun dried and grinded with a blender (Waring) and stored at room temperature in the dark. For making each batch of FL media, 4 g of the ground *Fucus* was stirred at 150 rpm for 2 hours with 100mL MilliQ water at room temperature. The mixture was then passed through 20 µm Steriflip filters (Millipore), diluted 2X or 4X with MilliQ water, and passed through a 0.22 µm PES filter (Corning). The stock solutions were stored in the dark at 4°C until use (up to 6 months). FL media was made from diluting the stock solution 20X (10X when stock solutions were 4X diluted) in a slightly modified version of the Tibbles-Rawlings minimal media [1, 2] and filtering again through a 0.22 µm PES filter (Corning). The concentration of dissolved organic material in FL media was determined by measuring the weight of dried material after lyophilizing 10 mL of the 10X stock solution.

Chemical characterization of FL

Glycosyl composition

The FL stock was lyophilized and the glycosyl composition was determined by the preparation of trimethylsilyl (TMS) methyl glycosides after methanolysis (1 M HCl-MeOH at 80 ºC; 18 h) in the presence of an internal standard (*myo*-inositol). The TMS derivatives were analyzed by GLC-MS [3] on a Hewlett-Packard HP5890 gas chromatograph equipped with mass selective detector 5970 MSD using an EC-1 fused silica capillary column (30 m × 0.25 mm I.D.), and temperature cycle at 80 ºC for 2 min, then ramp to 160 ºC at 20 ºC/min, and to 200 ºC at 2ºC/min, followed by an increase to 250 °C at 10 °C/min with an 11 min hold. Since the major monosaccharides found in the crude FL stock was sugar alcohol - mannitol, FL was dialyzed with a 1000Da cutoff Pur-A-Lyzer™ Mega Dialysis Kit (Sigma), and the dialyzed material was lyophilized and analyzed by another round of monosaccharide composition (TMS - methyl glycosides).

Glycosyl linkage

Before glycosyl linkage derivatization, the FL was treated overnight with Proteinase K (40°C), dialyzed (3,500 MWCO) at 4 ºC against several exchanges of dH_2O and freeze-dried, re-suspended in water, and precipitated three times with 9 vol. of EtOH. The insoluble precipitate was used for glycosyl linkage analysis. Due to the expected sulfation of the seaweed polysaccharides (fucoidan) in addition to neutral polysaccharides, the glycosyl linkage was performed on the desulfated and sulfated samples as previously described[4]. Briefly, to protect the sulfated polymers, the FL sample was passed through Dowex 50 cation exchange resin and converted to triethylammonium form before the linkage analysis. The sample was de-sulfated after passing through Dowex 50 cation exchange resin and conversion to pyridinium form followed by incubation in 89:10:1 DMSO–MeOH–pyridine (vol/vol/vol) for 4 h at 100 °C, and dialysis to remove the DMSO. Samples were freeze-dried and used for linkage analysis. Dry sulfated and de-sulfated FL extract were dissolved in 400 µL of dimethyl sulfoxide (DMSO) and left to stir for 24 h. Glycosyl linkage analysis of O-chain polysaccharides was obtained by GC-MS analysis of partially methylated alditol acetates (PMAA)[5] after 2h hydrolysis with 2M (v/v) TFA at 121ºC, overnight reduction with $NaBD_4$, and converted to alditol acetates with acetic anhydride in the presence of pyridine. GC-MS was performed on an HP-5890 GC interfaced to a mass selective detector 5970 MSD interfaced to a 5975C MSD (mass selective detector, electron impact ionization mode) PMAAs were resolved on a Supelco SP-

2331 bonded phase fused silica capillary column (30× 0.25 mm ID, Supelco) with temperature program: 60ºC for 1 min, then ramp to 170ºC at 27.5ºC/min, and to 235ºC at 4ºC/min with 2 min hold and finally at to 240ºC at 3ºC/min with 12-minute hold. The glycosyl linkages were determined based on the relative retention times to the standard of inositol and unique EI-MS fragmentation (https://glygen.ccrc.uga.edu/ccrc/specdb/ms/pmaa/pframe.html). The assessment and the localization of sulfation was based on a comparative neutral linkage analysis of sulfated vs. chemically *de*-sulfated polysaccharide. The significant loss of branched 2,3,4-substituted Fuc*p*, decrease of 2,3-substituted Fuc*p* and increase of 3,4-Fuc*p* and 4-Fuc*p* suggests that fucoidan polymer is likely sulfated at C-2 of the branching Fucose residue (either 2,3 or 2,3,4 substituted with side chain galactan). Not all of branching fucose residues may be sulfated. This is assessed by the presence of 3,4-linked Fuc*p* in the sample before de-sulfation (Fig. S1d).

Nuclear Magnetic Resonance Spectroscopy

The FL extract was exchanged twice with D_2O (99.9% atom %D; Aldrich) freeze-dried, and finally with D_2O 100% (Cambridge Isotope Laboratories, Inc) and the Proton NMR spectra were acquired at 70°C on a Varian 600-MHz Spectrometer equipped with a cold 6 mm probe. The water signal was suppressed by using standard presaturation experiment at approx. 4.3 ppm. The data were processed with MestReC/nova software (Mestrelab Research, Santiago de Compostela, Spain).

Sampling and incubation

Seawater was collected in 2 L aliquots in a 5×5 m area, and each aliquot was passed through a 70 µm mesh net until filling up a 50 L autoclaved screw-cap polypropylene container. The seawater inoculate was made from filtering 24 L of the collected seawater through a 5 µm filter (GE) onto 0.22 µm Sterivex filters (Millipore, SVGP01050) by using a peristaltic pump. To collect the surface bacteria community on sediment particles (Sediment inoculate), 40 g of sediment from the same location was placed in two separate 50 mL falcon tubes and mixed with 20 mL artificial seawater (Sigma Sea salts), and sonicated for 2 min in a bath sonicator (Cole-Parmer #8891). The supernatant was passed through a 5 µm filter (GE) onto 0.22 µm Sterivex filters using a peristaltic pump. The filters were peeled off the cartridge and equally divided into 6 pieces, with 3 pieces being used for inoculation of the FL media, 1 piece for negative control with no FL, and 2 pieces flash-frozen in an ethanol-dry ice bath for DNA extraction.

At each time point, biomass yield was determined by fixing the samples 1:1 with 1% formaldehyde containing SYBR Green (Thermo Fisher Scientific), and counting the number of cells via fluorescence activated cell sorting (FACS). Cultures (250 mL) were filtered through 0.22 µm PES filters (Corning), and the filtrates were used for dissolved organic carbon (DOC) measurements performed at the Nutrient Analytical Services Laboratory at the University of Maryland Center for Environmental Science with their standard operating procedure for DOC [6]. The filter paper was used for DNA extraction with the Qiagen Allprep Bacterial DNA/RNA/Protein Kit with a bead beating procedure. An 100 µL sample of each culture was also taken for collection of bacterial isolates (See isolate collection section below).

Isolate collection

For each time point, a 100 µL sample was diluted 10^5 and 10^6 -fold and plated onto 1.5% agar plates containing marine broth 2216 (Becton Dickinson, Difco #279110) undiluted or 10X diluted. Following 2 days of incubation at room temperature, 20-25 and 10-15 isolates were picked from each time point for each culture from undiluted and diluted media plates, respectively. Plates were inspected again after 7 days to selectively isolate slow growing isolates. All selected colonies were restreaked twice for purification, grown in deep well plates with liquid 2216/10X diluted 2216 broth for 48h, and saved at -

80°C in 20% glycerol. The final collection consisted of 1920 isolates, of which we selected 480 for taxonomic classification via Sanger sequencing the 16S rRNA V4 (515-786) region, and assigning the sequences using BLAST [7, 8] and the NCBI taxonomy database.

Reconstitution of succession with select isolates

For the 10 representative ASVs selected, one isolate per ASV (with 100% match in the 16S rRNA V4 region) was selected from the isolate collection for the reconstitution experiment. We tried to select strains that originated from the seawater inoculates and were isolated at the time when they were more abundant in the seawater inoculated succession. None of the taxonomically assigned isolates in our collection matched 100% in the 16S rRNA V4 region with the ASV Neptunomonas4, so we used a previous strain isolated from a *Fucus* associated community at the same location where the seawater inoculates were acquired.

Glycerol stocks of the 10 selected isolates were streaked onto 2216 plates, and single colonies were selected to grown overnight in liquid 2216 before transferred into pasteurized seawater collected from Canoe Beach on June 27th, 2018. The isolates underwent three dilution-growth cycles in the pasteurized seawater (1:100 dilutions every 5 days) before they were mixed to form a community of approximately 2×10^5 cells/mL matching the relative abundances measured in the seawater inoculate via 16S rRNA amplicon sequencing. ASVs (Celeribacter26, Vibrio2, and Arenibacter71) that were not detected in the seawater inoculate due to sequencing depth limitation were estimated to be at a concentration of 10 cells/mL. Also, because the Psychromonas9 strain died when growing in pasteurized seawater, we substituted it with the Psychromonas14 strain in the inoculum mix. The isolate-constituted community was grown in triplicate in 48-well deep-well blocks (Axygen) with 3 mL FL media in each well and shaking at 300 rpm on a tabletop shaker at room temperature. Time points were taken at 14, 24, 48, 72, 96, 120, 144, 168, 192, and 216 h. Biomass accumulation and community composition at each time point was determined using the standard protocols.

Standard Protocol for measuring biomass accumulation/cell density: Flow cytometry

Growth of all community and isolate cultures were tracked via flow cytometry. At each time point, 20 µL-500 µL subsamples of the cultures were obtained and fixed 1:10 with 0.8% paraformaldehyde (PFA, BeanTown Chemicals). The fixed samples were stained with staining media that contained either 1 µg/mL DAPI (Sigma) or 1X SYBR green for 30 min in the dark at room temperature before flow cytometry. All FACS measurements were performed using either a BD Canto or BD LSRFortessa Flow Cytometer with a high throughput sampler. Bacterial cells were gated by forward and side scatter as well as the intensity of DAPI staining (500 V laser with activation wavelength of 405 nm and collected through a 450/50 nm band-pass filter), or SYBR staining (400 V laser activation with activation wavelength of 488 nm and collected through a 530/30 nm band pass filter). Although gating was sometimes adjusted slightly depending on the specific daily laser shift of the flow cytometer, in general, signal points that were between $0\text{-}4\times10^4$ FSC-H, $40\text{-}4\times10^4$ SSC-H and showed more than 200 U blue (DAPI) /green (SYBR) fluorescence were counted as bacteria. Under this gating criteria, negative controls which contained only growth media usually had a baseline count of approximately 5×10^5 cells/mL; when taking measurements for samples, we included 3 negative control wells on each 96 well plate for FACS and used the average as the baseline count for that plate. As positive controls, the gating criteria was checked by comparing cell counts of isolates measured via flow cytometry (SYBR staining+ LSR Fortessa) to colony forming units on 2216 plates (Fig. S14a). Consistency of counts between different dyes and flow cytometers were also checked (Fig. S14b, c). Because the Cobetia10 isolate was found to stain less efficiently in DAPI compared to SYBR during stationary phase, care was taken to use

SYBR to stain isolate cultures of Cobetia10. An adjusting factor of 9.11(average of SYBR vs. DAPI for 9 biological replicates of stationary phase Cobetia10) was used to account for the discrepancy between the two stains when DAPI was used to stain Cobetia10 containing cultures at stationary phase.

Standard Protocol for determining community composition: DNA extraction, 16S rRNA gene library preparation and 16S rRNA gene amplicon data analysis

DNA extractions for all the model communities mixed with isolates were performed by filtering 100 µL of sample through MultiScreen HTS GV filter plates (0.22 µm, sterile, PVDF membrane, Millipore), incubating overnight with 1% Ready-Lyse Lysozyme (Epicentre) in TES buffer (10 mM Tris-HCl, 1 mM EDTA, 100 mM NaCl), and collecting the flow through after spinning the plates for 5 mins at 3000 rpm.

16S rRNA gene amplicon libraries (V4 hypervariable region, U515-E786) were prepared according to the a previously published method [9], with 0.3 mg/mL BSA (Molecular Biology grade, New England BioLabs) included in the first step PCR for better amplification efficiency. Samples were sequenced on a MiSeq System (PE 250+250, Illumina) at the BioMicro Center (Massachusetts Institute of Technology, Cambridge, MA).

All 16S rRNA gene amplicon libraries were processed according to a custom pipeline based on DADA2 [9, 10], resulting in amplicon sequencing variants (ASVs). All samples in the natural community successions were rarified to 3000 reads, while samples from the model communities were not rarified. Communities with less than 400 reads and taxa not seen in more than 3 reads in at least 10% of the samples were removed from the natural or model communities. For each sequencing batch, we included 2-5 negative controls which were growth media extracted, amplified and sequenced according to the same procedure as the samples. All negative controls had less than 400 reads, thus indicating that reads in our samples were truly from the accumulated biomass. Taxonomy for all sequence variants was assigned using the RDP database version 11.5 [11].

Bias introduced by DNA extraction, library preparation and amplicon sequencing was estimated by constructing mixed communities with defined composition with isolate cultures of known cell density (measured via FACS) and comparing the defined composition with that measured via 16S rRNA gene amplicon sequencing (Fig. S15). The method we used was similar in concept to a previously published method [12], and is described in detail below.

Bias correction for 16S rRNA libraries

The major assumption behind the method is that the relative bias between taxa remains constant in communities containing the same kinds of taxa (i.e., $\frac{FACS(A)/FACS(B)}{16S(A)/16S(B)}$ is constant in all communities of similar diversity containing taxon A and B):

$$\begin{pmatrix} b_1 & 0 & 0 & 0 \\ 0 & b_2 & 0 & 0 \\ 0 & 0 & \ddots & 0 \\ 0 & 0 & 0 & b_m \end{pmatrix} \times \begin{pmatrix} A_{1,1} & A_{1,2} & \cdots & A_{1,n} \\ A_{2,1} & A_{2,2} & \cdots & A_{2,n} \\ \vdots & \vdots & \vdots & \vdots \\ A_{m,1} & A_{m,2} & \cdots & A_{m,n} \end{pmatrix} \xrightarrow{Normalize\ columns\ to\ 100\%} \begin{pmatrix} O_{1,1} & O_{1,2} & \cdots & O_{1,n} \\ O_{2,1} & O_{2,2} & \cdots & O_{2,n} \\ \vdots & \vdots & \vdots & \vdots \\ O_{m,1} & O_{m,2} & \cdots & O_{m,n} \end{pmatrix}$$

Bias (**B**) matrix   Actual composition matrix (**A**)   Observed composition (**O**)

m ASVs   **m** ASVs × **n** Communities   **m** ASVs × **n** Communities

Thus, the bias of each ASV ($b_1, b_2 \cdots b_m$) can be calculated as the geometric mean of each row of the **O** Matrix divided by the corresponding row in the **A** matrix, element wise.

Biases were calculated separately for the 10 ASV model communities and 4 ASV model communities. In both cases, the biases among ASVs in a community were calculated using the above method by comparing the 0 h composition of the model communities estimated via FACS and that measured by 16S rRNA amplicon sequencing. The bias (**B**) matrix for the 10 ASV model community was used for correction of communities containing 10 or more ASVs, while the bias (**B**) matrix for the 4 ASV model community was used for all succession and coexistence data with 4 or less ASVs.

Burkholder diffusion assay

For the Burkholder diffusion assays, for each of the 10 selected isolates, a single colony growing on 2216 plates was transferred to 5 mL of marine broth 2216 and grown shaking at room temperature (25 °C) for 24 h. A soft agar lawn for each isolate, containing 150 µL of liquid culture in 2 mL of top agar (0.4%) was poured on a 1.5% bottom agar plate. To mimic the FL but also allow formation of a lawn, the media component of both the top agar and bottom agar was TRMM+ 0.1% FL+ 0.1% Fucose (Carl Roth) + 0.2% Mannitol (Carl Roth). Then 2 µl portions of the liquid cultures of all the 10 isolates, as well as a known highly antagonistic Vibrio strain, and a blank sample with only 2216 media were spotted on each isolate lawn in triplicates. The plates were incubated face up for 96 h at room temperature and examined daily for zones of inhibition. The FL used for the Burkholder diffusion assay was prepared according to the same procedure as described for all other experiments, except that the *Fucus* was commercially purchased (collected and sundried in Norway, Agora Market).

Selection of isolates for 4-strain model community

For selection of the 4-strain isolate community, we first eliminated Arenibacter71 and Alteromonas38 from the late collection since they were more functionally distant from the other isolates. We further eliminated the Psychromonas9 and Vibrio2 because they were almost functionally identical to Psychromonas14 and Vibrio1 and thus could be sufficiently represented by the latter group. Eventually, from the three remaining isolates in the early group, we selected Psychromonas14 and Vibrio1 as representatives because the directional functional overlaps (Psychromonas14 in spent media of Vibrio1 and vice versa) were more similar; in the late group we selected Cobetia10 and Neptunomonas4 because in the previous successions we observed a negative relationship between the two ASVs (Fig. S16).

Pre-culture preparation for isolate growth curves, spent media experiments, competition experiments and RNA-seq growth experiments

Selected isolates were streaked on marine broth 2216 1.5% agar plates and incubated at room temperature until visible colonies developed. Single colonies were mixed with pasteurized seawater and shaken overnight on a tabletop shaker at 300 rpm. The mixtures were then diluted 1:20 into FL media, grown for 24h, then diluted 1:50 into FL media again and grown for 18 h, and finally diluted into fresh FL or mannitol media so that the starting concentration of the growth curves would be approximately 10^5 cells/mL.

Spent media experiments

Pre-cultures for isolates in the spent-media experiment were prepared in the same way as for measuring the individual growth curves. For the 10 ASV × 10 ASV spent-media experiments, each isolate was grown in triplicate in 24 well blocks containing 3 mL FL media for 5 days before the spent-media was

harvested by filtration through MultiScreen HTS GV filter plates (0.22 µm, sterile, PVDF membrane, Millipore). The spent media of every ASV, as well as an "unspent" FL media, were inoculated 1:50 with all 10 ASVs respectively, and the cultures were grown in 96 well blocks at room temperature containing 220 µL spent media in each well, while shaking on a tabletop shaker at 300 rpm. Time points were taken at 0, 18, 36, 48, 72, 96 and 120 h to measure cell growth in each culture, and cell density at each time point was determined according to the standard protocol.

The spent-media experiment was also performed for a subset of strains (Psychromonas14, Vibrio1, Cobetia10 and Neptunomonas4, Arenibacter71) using a shorter time frame in both FL media and 0.0047% mannitol. All other procedures remaining the same, spent media was harvested for each isolate after 48 h of growth, and time points were taken at 0, 10, 18, 24, 32, and 48 h.

Succession of 4 strain model communities

A set of 8 communities from the first cycle of the 4-way competitions were also sampled at 0, 5, 10, 24, and 48 h for studying the short-term succession dynamics of the communities. These were the communities which the Neptunomonas4 were introduced at 1% starting density. At each time point, samples were taken and processed according to the standard protocol for measuring biomass accumulation and community composition (Supplementary methods). To accommodate this extra sampling, in the first cycle of the 4-way competitions, communities were grown in 2 mL cultures in 48 well deep well blocks.

Whole genome sequencing, assembly and functional annotation

Genomic DNA of all isolates was extracted from 200 µL liquid culture (OD_{600} ~0.4) with the Quick-DNA 96 kit (Cat #: D3012, ZYMO Research). Sequencing libraries were prepared according to a modified Illumina Nextera protocol [13] and pooled together for sequencing on a NextSeq 500 System (Illumina) for paired-end 75-bp reads (Broad Institute, Cambridge, MA). Reads for each isolate were first trimmed and filtered using Cutadapt 1.9.1 [14] and Sickle 1.33 [15] (pe -q 20 -l 50), and used as the input for de novo genome assembly via Spades v3.6.2 [16] (parameter: -careful). Contigs that were redundant or shorter than 480 bp were removed from the assembly.

Putative open reading frames (ORFs) in all assembled draft genomes were predicted using Prodigal via the PATRIC web server [17]. KEGG orthology (KO) numbers were assigned to the ORFs by using the KEGG Automatic Annotation Server [18] with the search program GHOSTX, and a manual set of 38 bacteria as the genes database (Table S2). Annotation cutoffs were defaults. Genes in each genome was assigned to KEGG pathway modules according to the KO list, using the R package KEGGREST[19]. Identification of polysaccharide lyases and glycosidic hydrolases were performed by searching all ORFs against the HMMs in the dbCAN database [20] with default settings. Only assignments that were consistent between two of the three methods (HMMER, diamond, Hotpep) were kept.

RNA-seq of selected isolates

RNA-seq was used to characterize the growth and metabolic processes for four strains in FL media. Because the RNA-seq data might be used to match future exo-metabolomic characterization of the isolate growth, we replaced the Tris HCl in our media (which could potentially interfere with the column for metabolomics) with 6 mM sodium bicarbonate. Functional overlap patterns of the four strains in this bicarbonate media with FL substrate are highly similar to the original TRMM (Fig. S17). Pre-cultures for the cultures used for RNA extraction were prepared according to the standard protocols. The cultures for RNA extraction were grown in 250 mL Erlenmeyer flasks containing 100 mL FL media, and inoculated

1:100 with the precultures in triplicates. Cultures were incubated at 23 °C with shaking at 300 rpm. Growth was determined by fixing and counting cells every 1-2 hours, and samples for transcriptomics were taken before and after the diauxic shifts for Psychromonas14 and Vibrio1, and in early and late log phase for Cobetia10 and Neptunomonas4. At each sampling point, a 30-40 mL subsample from each replicate was placed in 50 mL Falcon tubes and centrifuged for 5 min at 10000 × g at 4°C. In cases when the pellet was loose (Cobetia10 and Neptunomonas4) after the majority of the supernatant was removed, the pellet was re-dissolved in the leftover supernatant, transferred to 1.5 mL Eppendorf tubes, and centrifuged for 1.5 min at 15,000 × g at room temperature. Cell pellets from each sample were flash frozen in an ethanol dry ice bath and stored at −80 °C until RNA extraction.

RNA extraction was performed with the GenElute Total RNA Purification Kit [Sigma, standard protocol for gram-negative bacteria except for extended lysis time (20 mins)]. To minimize library prep costs/RNA extraction, we mixed the lysates of the isolates collected from the same growth phase (G1 or G2) in equal volumes after lysing them separately and continued with the standard RNA purification protocol.

Substrate Utilization Assay

We selected compounds that were representative substrates of the 8 expressed metabolic modules as follows: Histidine degradation (L-Histidine, Sigma), Leucine degradation (L-Leucine, Sigma), Tyrosine degradation (L-Tyrosine, Sigma), Nicotinate degradation (Nicotinic acid, Carl Roth), Pyrimidine degradation (Thymidine, Sigma), Benzonate degradation and Catechol ortho-cleavage (Sodium Benzoate, Sigma), and beta-oxidation (Oleic Acid, Fisher Scientific). Isolates tested were grown in marine 2216 broth overnight and 2 µL of culture was used to inoculate 200 µL TRMM media consisting of 0.1% mannitol and 0.1% of the selected substrate, or 200 µL TRMM media that only contained 0.1% mannitol as a baseline. The isolates were grown for 6 days in flat-bottom 96-well plates and O.D. 600 measurements were taken daily. The experiment was performed in triplicates for each isolate – substrate pair, and a difference of >0.15 in the maximum O.D. measured for the 0.1% mannitol + 0.1% selected substrate vs. the baseline 0.1% mannitol media was considered as positive growth.

Fucoidan growth curves

Growth curves of the isolates in 0.1% Fucoidan were prepared same as above, expect that only 3 time points were taken over the course of 48 hours. The Fucoidan used was purified from *Fucus versiculosis* (>95%, Sigma, F8190). Given the relatively low purity of the substrate, only growth to at least 10^8 cells/mL was considered as positive growth on Fucoidan.

References for Supplementary Methods

1. Datta MS, Sliwerska E, Gore J, Polz MF, Cordero OX. Microbial interactions lead to rapid micro-scale successions on model marine particles. *Nat Commun* 2016; **7**: ncomms11965.

2. Tibbles BJ, Rawlings DE. Characterization of Nitrogen-Fixing Bacteria from a Temperate Saltmarsh Lagoon, including Isolates That Produce Ethane from Acetylene. *Microb Ecol* 1994; **27**: 65–80.

3. York WS, Darvill AG, McNeil M, Stevenson TT, Albersheim P. Isolation and characterization of plant cell walls and cell wall components. *Methods in Enzymology*. 1986. Academic Press, pp 3–40.

4. Kariya Y, Watabe S, Kyogashima M, Ishihara M, Ishii T. Structure of fucose branches in the glycosaminoglycan from the body wall of the sea cucumber Stichopus japonicus. *Carbohydr Res* 1997; **297**: 273–279.

5. Ciucanu I, Kerek F. A simple and rapid method for the permethylation of carbohydrates. *Carbohydr Res* 1984; **131**: 209–217.

6. Laboratory Methods. *University of Maryland Center for Environmental Science*. https://www.umces.edu/nasl/methods. Accessed 31 Oct 2022.

7. Camacho C, Coulouris G, Avagyan V, Ma N, Papadopoulos J, Bealer K, et al. BLAST+: architecture and applications. *BMC Bioinformatics* 2009; **10**: 421.

8. Altschul SF, Gish W, Miller W, Myers EW, Lipman DJ. Basic local alignment search tool. *J Mol Biol* 1990; **215**: 403–410.

9. Yu X, Polz MF, Alm EJ. Interactions in self-assembled microbial communities saturate with diversity. *ISME J* 2019; **13**: 1602.

10. Callahan BJ, McMurdie PJ, Rosen MJ, Han AW, Johnson AJA, Holmes SP. DADA2: High-resolution sample inference from Illumina amplicon data. *Nat Methods* 2016; **13**: 581.

11. Cole JR, Wang Q, Fish JA, Chai B, McGarrell DM, Sun Y, et al. Ribosomal Database Project: data and tools for high throughput rRNA analysis. *Nucleic Acids Res* 2014; **42**: D633–D642.

12. McLaren MR, Willis AD, Callahan BJ. Consistent and correctable bias in metagenomic sequencing experiments. 2019. Bioinformatics.

13. Baym M, Kryazhimskiy S, Lieberman TD, Chung H, Desai MM, Kishony R. Inexpensive Multiplexed Library Preparation for Megabase-Sized Genomes. *PLOS ONE* 2015; **10**: e0128036.

14. Martin M. CUTADAPT removes adapter sequences from high-throughput sequencing reads. *EMBnet.journal* 2011; **17**.

15. Joshi NA, Fass JN. (2011). Sickle: A sliding-window, adaptive, quality-based trimming tool for FastQ files _(Version 1.33) [Software]. Available at https://github.com/najoshi/sickle.

16. Bankevich A, Nurk S, Antipov D, Gurevich AA, Dvorkin M, Kulikov AS, et al. SPAdes: A New Genome Assembly Algorithm and Its Applications to Single-Cell Sequencing. *J Comput Biol* 2012; **19**: 455–477.

17. Wattam AR, Abraham D, Dalay O, Disz TL, Driscoll T, Gabbard JL, et al. PATRIC, the bacterial bioinformatics database and analysis resource. *Nucleic Acids Res* 2014; **42**: D581–D591.

18. Moriya Y, Itoh M, Okuda S, Yoshizawa AC, Kanehisa M. KAAS: an automatic genome annotation and pathway reconstruction server. *Nucleic Acids Res* 2007; **35**: W182–W185.

19. Tenenbaum D, Maintainer BP. KEGGREST: Client-side REST access to the Kyoto Encyclopedia of Genes and Genomes (KEGG). 2022.

20. Zhang H, Yohe T, Huang L, Entwistle S, Wu P, Yang Z, et al. dbCAN2: a meta server for automated carbohydrate-active enzyme annotation. *Nucleic Acids Res* 2018; **46**: W95–W101.
